# Antagonistic chemical coupling in self-reconfigurable host–guest protocells

**DOI:** 10.1038/s41467-018-06087-3

**Published:** 2018-09-07

**Authors:** Nicolas Martin, Jean-Paul Douliez, Yan Qiao, Richard Booth, Mei Li, Stephen Mann

**Affiliations:** 1 Centre for Protolife Research and Centre for Organized Matter Chemistry, School of Chemistry, University of Bristol, Bristol, BS8 1TS UK; 2 UMR 1332, Biologie et pathologie du fruit, INRA, University of Bordeaux, Villenave d’Ornon, France; 3 State Key Laboratory of Polymer Physics and Chemistry, Institute of Chemistry, Chinese Academy of Sciences, Beijing, 100190 China

## Abstract

Fabrication of compartmentalised chemical systems with nested architectures and biomimetic properties has important implications for controlling the positional assembly of functional components, spatiotemporal regulation of enzyme cascades and modelling of proto-organelle behaviour in synthetic protocells. Here, we describe the spontaneous capture of glucose oxidase-containing proteinosomes in pH-sensitive fatty acid micelle coacervate droplets as a facile route to multi-compartmentalised host–guest protocells capable of antagonistic chemical and structural coupling. The nested system functions co-operatively at low-substrate turnover, while high levels of glucose give rise to pH-induced disassembly of the droplets, release of the incarcerated proteinosomes and self-reconfiguration into spatially organised enzymatically active vesicle-in-proteinosome protocells. Co-encapsulation of antagonistic enzymes within the proteinosomes produces a sequence of self-induced capture and host–guest reconfiguration. Taken together, our results highlight opportunities for the fabrication of self-reconfigurable host–guest protocells and provide a step towards the development of protocell populations exhibiting both synergistic and antagonistic modes of interaction.

## Abstract (web summary)

Multi-compartmentalised soft micro-systems are used as models of synthetic protocells. Here, the authors developed nested host–guest protocell constructs capable of self-reconfiguration in response to changes in pH generated by antagonistic modes of enzyme-mediated coupling.

## Introduction

Micro-compartmentalised chemical systems endowed with biomimetic functions offer a range of new approaches to synthetic colloidal objects with cell-like behaviours (protocells)^1–3^. Single-compartment protocellular platforms include lipid^4–6^ and polymer^7^ vesicles, inorganic nanoparticle-stabilised colloidosomes^8^, microcapsules delimited by a monolayer shell of cross-linked protein–polymer nano-conjugates^9^ (proteinosomes) and membrane-free liquid micro-droplets prepared by associative^10^ (coacervates) or segregative^11^ (aqueous two-phase systems) liquid–liquid phase separation. The integration of functional components within these constructs has paved the way towards the elucidation of the physical and chemical basis of enzymatic catalysis in confined^12,13^ or crowded^14–16^ environments, encapsulated gene-mediated cell-free protein expression^17,18^, artificial cytoskeleton assembly^19,20^, protein folding^21^, selective membrane gating^8^, motility^22^ and membrane growth and division^23–25^.

Recent studies have extended the design strategies underpinning these synthetic protocell models to increase their functional complexity by reproducing aspects of cellular multi-compartmentalisation^26,27^. The construction of synthetic multi-compartment micro-architectures often involves sophisticated devices or processing techniques and has been primarily circumscribed to the nesting of a single protocell type. For instance, proteinosome-in-proteinosome microstructures were prepared by sequential Pickering emulsion processing^28^, and liposome-in-liposome constructs (vesosomes) by using lipid film hydration procedures^29^ or microfluidic approaches involving the dewetting of double emulsion templates^30^. Similarly, polymersome-in-polymersome nested assemblies have been fabricated by emulsion-centrifugation methods^31,32^, microfluidics^33^, two-stage double emulsion processing^34^ or sequential deposition of polymersomes and polymer multi-layers onto sacrificial micro-particle templates^35^. Multi-compartmental hydrogel particles have been obtained by microfluidics^36^, and hydrogel matrices employed to house lipid-stabilised water droplets in oil droplet nested structures^37^. Recently, the spontaneous formation of proteinosome-in-proteinosome structures by a single-step double emulsion method was demonstrated^38^, and colloidosome-in-colloidosome constructs were obtained by spontaneous engulfment^39^. In contrast to the above studies on single protocell types, there has been relatively few investigations on the integration of multiple types of protocells within nested constructs except for pioneering work on liposome-in-polymersome^40–42^ and coacervate-in-liposome^11,43,44^ micro-architectures.

To date, the above-mentioned nested micro-architectures have essentially been used for the positional assembly and spatial confinement of different functional components within proto-organelles that are synergistically coupled for the programmed release of biomolecules under specific stimuli^28,42^, inter-compartmental delivery of substrates^39^, spatiotemporal control of enzyme cascade reactions^32,35^ or the localisation of cell-free protein expression modules^44^. In contrast, the fabrication of nested compartments involving different types of protocells capable of being dynamically restructured to produce higher-order behaviours has received little attention. In this regard, the investigation of collective properties in synthetic protocell populations is an emerging paradigm that has recently been exploited for the design and conception of artificial predatory-prey^45^ and phagocytosis-inspired^39^ behaviours.

In this paper, we address the challenge of how to design multi-compartmentalised heterogeneous soft matter micro-systems capable of synergistic or antagonistic modes of chemical coupling, and show how non-mutual interactions give rise to self-reconfigurable host–guest synthetic protocells displaying self-induced structural and functional reorganisation. The hybrid constructs consist of two primary protocell types arranged in a nested microstructure derived from the spontaneous capture and confinement of glucose oxidase (GOx)-containing guest proteinosomes within individual horseradish peroxidase (HRP)-containing fatty acid micelle coacervate host micro-droplets. Integration of the primary protocells results in synergistic or antagonistic modes of interaction depending on the level of GOx activity within the host–guest construct. At low-substrate turnover, the proteinosome-in-coacervate system functions co-operatively as a spatially coordinated enzyme cascade reaction, while high turnovers give rise to proteinosome-mediated pH-induced disassembly of the host coacervate droplets and release of the incarcerated proteinosomes. Restructuring of the nested micro-architecture transforms the micelle coacervate phase into fatty acid vesicles that self-assemble along with certain molecular payloads within the proteinosomes to produce vesicle-in-proteinosome protocell micro-architectures. We use cell-sorting techniques and optical and fluorescence microscopy to monitor the population dynamics and restructuration in the protocell consortia. Finally, stepwise triggering of antagonistic enzymes (urease and GOx) co-encapsulated within the proteinosomes is used to initiate self-induced capture and host/guest reconfiguration via a sequence of spatially dependent proteinosome-mediated vesicle-to-coacervate and coacervate-to-vesicle transformations. Taken together, our results highlight opportunities for the spontaneous construction of self-reconfigurable host–guest protocells, and provide a step towards the development of protocell populations exhibiting both synergistic and antagonistic modes of interaction.

## Results

### Spontaneous capture of proteinosomes in coacervate droplets

**Fig. 1:**
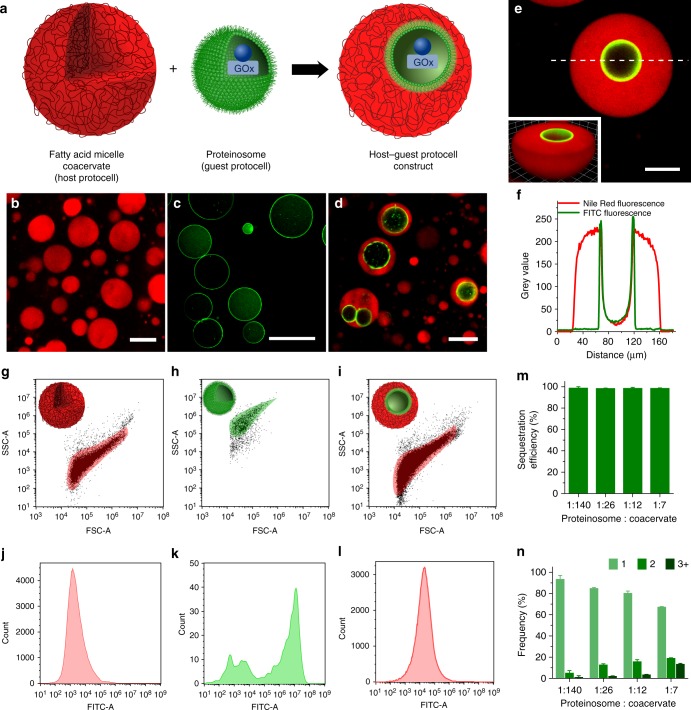
Spontaneous capture of proteinosomes in fatty acid micelle coacervate micro-droplets. **a** Scheme illustrating formation of host–guest protocells in water via the spontaneous incarceration of single or multiple GOx-containing BSA–NH_2_/PNIPAAm cross-linked proteinosomes (green) within individual fatty acid micelle coacervate micro-droplets (red). **b**-**d** Confocal fluorescence microscopy images; **b** myristic acid micelle coacervate droplets doped with Nile Red (red fluorescence), **c** GOx-containing FITC-labelled proteinosomes (green fluorescence), and **d** host–guest protocells prepared by mixing the proteinosomes and coacervate droplets at a number density ratio of 1:12. **e**, **f** Confocal fluorescence microscopy image of a single host–guest protocell (**e**) and corresponding line profile (white dashed line in **e**) of green and red fluorescence intensity across the proteinosome-in-coacervate microstructure (**f**). Note the localisation of green fluorescence specifically with the FITC-labelled proteinosome membrane, and absence of the coacervate phase inside the proteinosome. **g**–**i** FACS-derived 2D dot plots of side-scattered light (SSC) vs. forward-scattered light (FSC) for single populations of fatty acid micelle coacervate droplets (**g**), GOx-containing FITC-labelled proteinosomes (**h**) and GOx-containing proteinosome-in-coacervate protocells (*n* *=* 20,000; proteinosome : coacervate droplet number density ratio, 1:12) (**i**). Incarceration of the proteinosomes within the larger micelle coacervate droplets generates a scattering distribution almost identical to the initial population of host coacervate protocells. **j**–**l** Corresponding histograms of the green fluorescent signal (FITC-A) for samples shown in **g**–**i**; low and high levels of fluorescence are recorded in the micelle coacervate (**j**) and proteinosome (**k**) single populations, respectively; an intermediate level of green fluorescence is observed for the nested microstructure of the host–guest protocells (**l**). **m** Plot showing very high levels of sequestration efficiency (percentage of proteinosomes internalised by micelle coacervate droplets) across a range of proteinosome : coacervate droplet number ratios. Error bars represent the standard deviation of the sequestration efficiency (*n* = 3). **n** Plot showing statistical distribution of number of proteinosomes captured per coacervate droplet at various proteinosome : coacervate droplet number ratios. Error bars represent the standard deviation of the number of proteinosomes per coacervate droplet (*n* = 3)

Synthetic host–guest protocells comprising a nested microstructure of GOx-containing guest proteinosomes housed within individual fatty acid micelle coacervate host micro-droplets were produced spontaneously by mixing aqueous suspensions of the two types of primary protocells (Fig. 1a). The pH-sensitive coacervate micro-droplets consisted of an entangled network of elongated fatty acid micelles^46^, and were prepared by addition of a twofold molar excess of guanidinium counterions to a micellar dispersion of myristic acid (CH_3_(CH_2_)_12_COOH) at pH 9.5^46,47^. The membrane-free droplets exhibited high optical contrast and were readily imaged by fluorescence microscopy when doped with the hydrophobic dye Nile Red (Fig. 1b). The images showed discrete droplets with a uniformly distributed red fluorescence and diameters between 5 and 200 μm depending on the degree of coalescence (Supplementary Fig. 1). In contrast, the water-filled proteinosomes consisted of a semipermeable nanometre-thin membrane of covalently cross-linked amine-modified bovine serum albumin (BSA–NH_2_)/poly(*N*-isopropylacrylamide) (PNIPAAm) nano-conjugates^9,12^. Proteinosomes with fluorescein isothiocyanate (FITC)-labelled protein–polymer shells showed low-optical contrast, but were readily imaged by confocal fluorescence microscopy, which revealed spherical, non-aggregated membrane-bounded micro-compartmentalised colloidal objects (Fig. 1c and Supplementary Fig. 2) with diameters between 10 and 80 μm (Supplementary Fig. 1). Non-fluorescent proteinosomes loaded with FITC-labelled GOx exhibited a uniform green fluorescence throughout their interior (Supplementary Fig. 2), which was consistent with the retention and homogeneous distribution of the entrapped enzyme.

Mixing aqueous dispersions of the proteinosomes and micelle coacervate droplets at pH 9.5 and 32 °C, and at a range of proteinosome : coacervate droplet number density ratios (1:140, 1:26, 1:12 and 1:7) resulted in spontaneous capture and confinement of single or multiple proteinosomes within individual coacervate droplets. Optical and confocal fluorescence microscopy imaging of samples prepared with FITC-labelled proteinosomes and Nile Red-doped micelle coacervate droplets revealed a distinct proteinosome-in-coacervate nested structure (Fig. 1d). The engulfed proteinosomes remained structurally intact and spherical in shape, and were able to move freely within the surrounding micelle coacervate matrix, but remained irreversibly trapped. Fluorescence intensity line profiles recorded across the proteinosome-in-coacervate architecture revealed an absence of red fluorescence in the water-filled lumen of the incarcerated proteinosomes (Fig. 1e, f), indicating that the elongated fatty acid micelles did not diffuse across the semipermeable BSA–NH_2_/PNIPAAm membrane. Moreover, confocal fluorescence microscopy images of host–guest protocells prepared from populations of non-fluorescently labelled proteinosomes containing FITC-labelled GOx indicated that the enzyme was retained within the water-filled lumen of the guest proteinosomes for at least 2 h after confinement within the micelle coacervate droplets (Supplementary Fig. 3).

Sequestration of the proteinosomes by the micelle coacervate droplets was also analysed statistically by fluorescence-activated cell sorting (FACS). Individual populations of non-labelled micelle coacervate droplets (Fig. 1g) and FITC-labelled proteinosomes (Fig. 1h) gave 2D dot plots of side-scattered (SSC) vs. forward-scattered light that were clearly distinguishable. The proteinosomes exhibited higher SSC values, presumably due to an increased granularity, as previously reported^45^. As expected, the green fluorescence mean signal measured for the FITC-tagged proteinosome population was several orders of magnitude greater than that associated with the non-fluorescent coacervate micro-droplets (≈10^3^) (Fig. 1j, k). After mixing the two primary protocell populations at a final proteinosome : coacervate number density ratio of 1:12, only one population with a mean fluorescence intensity value of ≈10^4^ was observed in the 2D dot plot (Fig. 1i, l), consistent with formation of a host–guest proteinosome-in-coacervate nested microstructure. Statistical image analysis using fluorescence microscopy indicated that the sequestration efficiency was almost 100% at a range of number ratios (Fig. 1m). Empty micelle coacervate droplets or droplets containing only one proteinosome were preferentially formed at low proteinosome : coacervate number density ratios, while increasing the relative numbers of proteinosomes resulted in the formation of host–guest protocells containing single and multiple proteinosomes (Fig. 1n).

The highly efficient sequestration of the proteinosomes by coacervate micro-droplets was attributed to the preferential solubility of the PNIPAAm-rich surface of the proteinosomes in the hydrophobic fatty acid droplet environment. This was confirmed by the significantly enhanced partition coefficient of the water soluble BSA–NH_2_/PNIPAAm nanoconjugate building blocks at 32 °C (*K* = 34 ± 0.3, mean ± s.d., *n* = 3) compared to that of native BSA (*K* = 1.8 ± 0.5, mean ± s.d., *n* = 3) or BSA–NH_2_ (*K* = 7.6 ± 2.6, mean ± s.d., *n* = 3). As a consequence, adventitious interactions between the two types of protocells resulted in wetting of the proteinosomes by the micelle coacervate droplets such that internalisation of proteinosomes by large single droplets occurred spontaneously in a single step (Supplementary Movie 1 and Supplementary Fig. 4).

### Synergistic and antagonistic behaviour

**Fig. 2:**
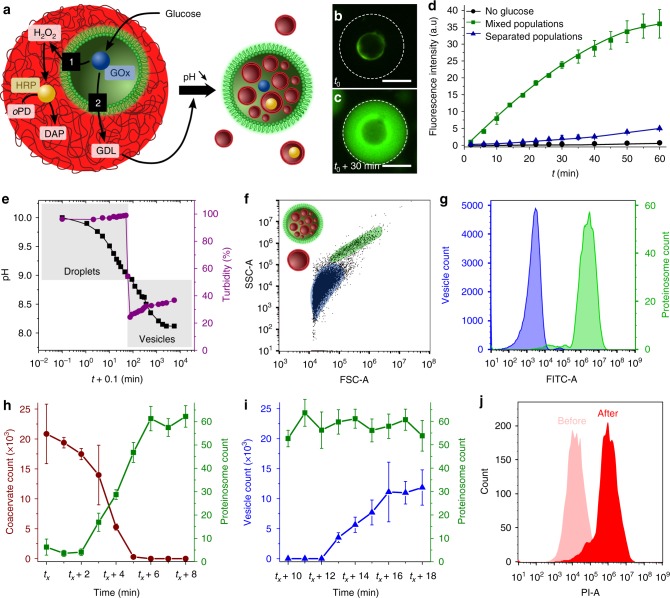
Synergistic and antagonistic behaviour in synthetic host–guest protocells. **a** Graphic showing FITC-labelled proteinosome (green) containing GOx (blue circle) internalised by a single pH-responsive fatty acid micelle coacervate droplet (red) loaded with horseradish peroxidase (HRP, yellow circle) to form a nested proteinosome-in-coacervate structure capable of synergistic (path 1) or antagonistic (path 2) chemical coupling. *o*PD *ortho*-phenylenediamine; DAP 2,3-diaminophenazine; GDL glucono-δ-lactone. **b**, **c** Fluorescence microscopy images showing a single proteinosome-in-coacervate protocell immediately after addition of glucose (1 mM, *t*_0_) (**b**), and after 30 min reaction time (**c**). **d** Time-dependent changes in fluorescence intensity associated with the formation of DAP in host–guest protocells produced as described in **a** (green squares), or in spatially separated protocell populations in the presence (blue triangles) or absence (control; black dots) of 1 mM glucose (see Methods). Error bars represent the standard deviation of the fluorescence intensity (*n* = 3). **e** Time-dependent plot showing gradual decrease in pH (black squares) and associated rapid decrease in turbidity (purple circles) due to vesicle formation at pH 8.9 for a population of GOx-containing proteinosome-in-micelle coacervate protocells (number density ratio = 1:12) incubated in glucose (*t* = 0, 10 mM). **f** FACS-derived 2D dot plots recorded 120 min after addition of glucose (10 mM) to a suspension of FITC-labelled GOx-containing proteinosome-in-coacervate host–guest protocells (number density ratio = 1:12) showing coexistence of a binary population of free myristic acid vesicles (blue domain) and proteinosomes containing fatty acid vesicles (green domain). FSC forward-scattered light; SSC side-scattered light. **g** Histograms of green fluorescence signals (FITC-A) recorded 120 min after addition of 10 mM glucose to FITC-labelled GOx-containing proteinosome-in-coacervate protocells; plots for unlabelled myristic acid vesicles dispersed in the bulk phase (blue, low fluorescence) and proteinosomes containing fatty acid vesicles (green, high fluorescence) are shown. **h** Plots showing population dynamics associated with the disassembly of GOx-containing FITC-labelled proteinosome-in-micelle coacervate droplets (red, number ratio = 1:12) and release of the entrapped proteinosomes (green). Counts were determined from the FACS-derived 2D dot plots, averaged every 5 s, and the standard deviation calculated accordingly. **i** Plots as derived in **h** but recorded over a period of 10 min and beginning from *t*_*x*_ + 10 min showing constant and increasing vesicle-in-proteinosome (green) and free vesicle (blue) populations, respectively. **j** FACS-derived red fluorescence intensity (PI-A) histograms determined from proteinosome-gated 2D dot plots recorded on a single GOx-containing, non-labelled proteinosome population before mixing with Nile Red-stained micelle coacervate droplets, and 120 min after addition of 10 mM glucose to a dispersion of GOx-containing proteinosome-in-coacervate protocells

Integration of the primary protocells into discrete nested microstructures was exploited for the design of enzymatically active host–guest protocells capable of displaying mutual or non-mutual behaviour depending on the level of GOx activity within the entrapped proteinosomes. As glucose oxidation gave rise to the in situ generation of both hydrogen peroxide and glucono-δ-lactone (GDL), we used these products, respectively, to induce synergistic or antagonistic modes of operation in the host–guest protocells. Under low-glucose concentrations (1 mM), a H_2_O_2_-mediated spatially coordinated enzyme cascade reaction was established by sequestration of HRP (*K* = 18 ± 1, mean ± s.d., *n* = 3) and encapsulation of GOx specifically within the host micelle coacervate or guest proteinosomes, respectively (Fig. 2a, path 1). *Ortho-*phenylenediamine (*o*PD, substrate for HRP) was also sequestered within the micelle coacervate phase, and the GOx/HRP cascade reaction induced by addition of glucose to the external medium at a concentration ≤1 mM. Corresponding fluorescence microscopy images of individual proteinosome-in-coacervate protocells showed a progressive increase in green fluorescence preferentially within the coacervate droplet due to formation of the oxidised product 2,3-diaminophenazine (DAP) (Fig. 2b, c). This was consistent with a time-dependent increase in the fluorescence intensity of the reaction system, and confirmed that the enzymatically active host and guest protocell populations operated synergistically (Fig. 2d). The initial rate of reaction monitored over 60 min at 32 °C was 20 μM min^−1^. In contrast, very slow rates of enzymatic activity (1.5 μM min^−1^) were observed when the cascade reaction was performed in populations of proteinosomes and micelle coacervate droplets that were spatially separated by a dialysis membrane permeable to H_2_O_2_ (Fig. 2d) (see Methods). The lower initial reaction rate was attributed to the increased time required for H_2_O_2_ to diffuse from the proteinosomes to the coacervate micro-droplets compared with diffusion within the nested host–guest configuration.

Alternatively, enzyme-containing host–guest protocells exhibiting non-mutual modes of behaviour were established at high-glucose concentrations (10 mM). Under these conditions, the high levels of GDL production resulted in a pH decrease sufficient to disassemble the fatty acid micelle coacervate host, release the incarcerated proteinosomes and induce the self-assembly of fatty acid vesicles (Fig. 2a, path 2). Typically, the pH decreased from around 10 to a constant value of approximately 8 after 20 h, and was accompanied by a rapid decrease in turbidity from 100 to ca. 30% at pH 8.9 (Fig. 2e and Supplementary Fig. 5). The latter was attributed to the proteinosome-mediated disassembly of the micelle coacervate host droplets into a heterogeneous population of unilamellar and multi-lamellar myristic acid vesicles^47^ with hydrodynamic diameters between 0.05 and 6 μm (Supplementary Fig. 6). Our results point towards a synchronised disassembly of the coacervate micro-droplets driven by a decrease in the environmental pH below a critical value of 8.9, which was attributed to the production of a sufficiently high concentration of gluconic acid to counter-balance the buffering effect of the fatty acid molecules. The time required to reach the threshold pH value of 8.9 decreased exponentially as the proteinosome : coacervate number density ratio was increased, indicating that it was possible to temporally programme the proteinosome guest-mediated disassembly of the host coacervate droplets (Supplementary Fig. 7). In contrast, when the host–guest protocells were exposed to glucose concentrations below 1 mM, the pH remained above the threshold value of 8.9 and no changes in turbidity were observed (Supplementary Fig. 7). Under these conditions, the nested architecture was preserved and the enzymatically active host–guest system functioned synergistically rather than antagonistically.

The rudimentary parasite-like behaviour of the enzymatically active guest proteinosomes was monitored statistically by FACS. 2D dot plots recorded 120 min after addition of 10 mM glucose to a dispersion of the nested structures indicated that the single population of proteinosome-in-coacervate nested protocells (Fig. 1i) was replaced by a binary population (Fig. 2f), consisting of a mixture of fatty acid vesicles exhibiting a low-green fluorescence signal (Fig. 2g and Supplementary Fig. 8), and FITC-labelled proteinosomes with strong green fluorescence (Fig. 2g). Time-dependent FACS experiments recorded within 10 min of glucose addition to a host–guest protocell suspension initially prepared at pH 9.1 showed a progressive disappearance of the fatty acid micelle coacervate population, release of the captured proteinosomes and formation of myristic acid vesicles (Supplementary Fig. 9). The initial (10 min) population dynamics were associated, respectively, with a coupled decrease and increase in the counts arising from the disassembly of the proteinosome-in-coacervate droplets and concomitant release of the incarcerated proteinosomes (Fig. 2h). After 10 min of glucose addition, the proteinosome count became constant, while counts for the fatty acid vesicles progressively increased from zero to a plateau value within a transitional period of 16 min (Fig. 2i). The gradual formation of the vesicles was clearly observed in the 2D dot plots (Supplementary Fig. 9) and gave rise to a corresponding slow increase in turbidity (Fig. 2e). Significantly, studies using GOx-containing host–guest protocells assembled from Nile Red-stained micelle coacervate droplets confirmed that a population of the fatty acid vesicles were trapped specifically within the released proteinosomes (Fig. 2j).

The above results demonstrate that steps towards antagonistic modes of behaviour can be introduced into proteinosome-in-coacervate nested protocells by internally triggered enzyme-mediated acidification associated with high-glucose turnover by the guest proteinosomes. The high sensitivity of the fatty acid micelle coacervate host droplets to protonation results in guest-mediated disassembly, escape of the entrapped proteinosomes and fatty acid vesicle formation, indicating that the nested microstructures are capable of self-induced reconfiguration.

### Self-induced disassembly and transformation

**Fig. 3:**
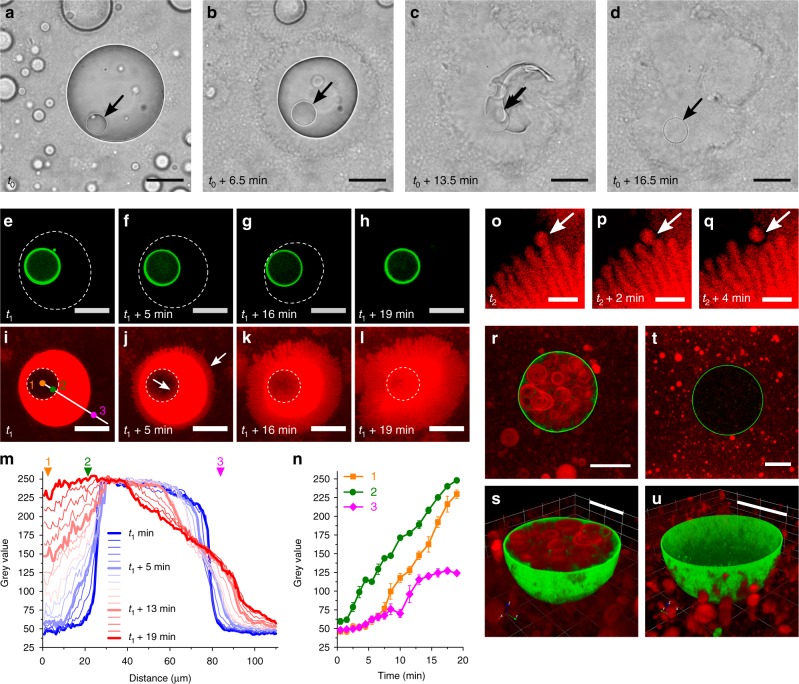
Disassembly and transformation in self-reconfigurable host–guest protocells. **a**–**d** Time series of optical microscopy images showing enzyme-mediated disassembly and transformation of an individual GOx-containing proteinosome/myristic acid micelle coacervate droplet in the presence of glucose (10 mM; time *t*_0_). Arrow shows location of the single guest proteinosome (low-optical contrast) that remains intact as the host micelle coacervate droplet (high optical contrast) is progressively disassembled and transformed into a corona of elongated fatty acid vesicles (low-optical contrast). The proteinosome is released as an intact micro-capsule (Supplementary Movie 2); scale bars, 50 μm. **e**–**l** As for **a**–**d**, but imaged by confocal fluorescence microscopy showing presence of intact FITC-labelled guest proteinosome at time *t*_1_ (green ring in **e**–**h**, dashed line in **i**–**l**) within a disassembling Nile Red-doped micelle coacervate droplet (dashed line in **e**–**h**, red object in **i**–**l**). Progressive outgrowth of red fluorescent elongated protrusions occurs from the surface of the deconstructing micelle coacervate droplet, along with inward contraction into the proteinosome interior (white arrows) (Supplementary Movie 3); scale bars, 50 μm. Numbers and white line in **i** refer to line profile and specific locations used to measure changes in red fluorescence (see **m**, **n**). **m**, **n** Spatial- (**m**) and time (**n**)-dependent changes in red fluorescence intensities for the host–guest protocell shown in **i**–**l**. Line profiles are shown in **m** (white line in **i**), and intensity values measured at three specific locations (1, 2, 3 in **i**) are shown in **n**. Error bars in **n** represent the standard deviation of the fluorescence intensity. **o**–**q** High-magnification confocal fluorescence microscopy images showing detachment of spherical fatty acid vesicles at the end of elongated protrusions formed at the droplet/water interface over a period of 4 min (white arrows); scale bars, 10 μm. **r**, **s** 2D confocal microscopy image (**r**) and 3D reconstruction (**s**) of a single proteinosome containing multiple fatty acid vesicles after enzyme-mediated self-induced transformation of a FITC-labelled proteinosome/Nile Red-doped micelle coacervate nested protocell. **t**, **u** As for **r**, **s**, but after a more rapid decrease in pH (addition of HCl) showing absence of vesicle sequestration in the reconstructed hybrid protocell; scale bars, 20 μm

Optical and confocal fluorescence microscopies were used to gain insight into the structural changes accompanying the self-induced disassembly and transformation of individual host–guest protocells. Time-dependent optical microscopy observations of nested microstructures produced by the spontaneous capture of GOx-containing FITC-labelled proteinosomes within Nile Red-doped micelle coacervate droplets and exposed to 10 mM glucose showed a progressive decrease over ca. 15 min in droplet size combined with the outgrowth of an optically transparent and birefringent corona of elongated protrusions (Fig. 3a–d, Supplementary Movie 2 and Supplementary Fig. 10). Similar observations were made by confocal fluorescence microscopy, which indicated that the guest proteinosomes remained unchanged in shape and size as the host protocell was dismantled by acidification (Fig. 3e–l and Supplementary Movie 3). Fluorescence intensity line profiles recorded at various time intervals during the coupled disassembly and transformation processes showed a progressive increase in red fluorescence associated with Nile Red penetration into the incipient coronal layer (Fig. 3m, n), indicating that the elongated protrusions were multi-lamellar tubules of partially protonated myristic acid. High-magnification confocal fluorescence microscopy images revealed that fatty acid spherical vesicles gradually detached from the ends of the elongated protrusions (Fig. 3o–q). Structural reorganisation of the fatty acid micelle coacervate droplet was also associated with the onset of Nile Red fluorescence in the interior of the guest proteinosome (Fig. 3m, n). With time, the corona of tubules was completely transformed into a bulk dispersion of vesicles such that the guest GOx-containing proteinosomes were released into the aqueous environment. Subsequent fluorescence microscopy images showed the presence of multiple fatty acid vesicles also within the intact proteinosomes (Fig. 3r, s and Supplementary Fig. 11), consistent with FACS studies (Fig. 2j).

As the fatty acid vesicles remained encapsulated within the proteinosomes, the results suggested that the protein–polymer membrane was permeable to solubilized myristic acid molecules released from the disassembling micelle coacervate droplet at intermediate pH values. Control experiments performed by rapidly decreasing the pH to a value of 8.5 by addition of aqueous HCl (Supplementary Fig. 11) revealed an absence of vesicles inside the proteinosomes (Fig. 3t, u), while a stepwise decrease of the pH to 8.5 by gradual addition of HCl (Supplementary Fig. 11) resulted in the formation and accumulation of trapped vesicles. In all experiments, vesicles were observed only if the pH was decreased below a value of 8.9.

Taken together, the experiments indicated that accumulation of vesicles inside the proteinosomes was facilitated by the slow and sustained pH decrease associated with GOx activity, as well as the high local concentration of free fatty acid molecules generated during disassembly and transformation of the nested protocells. These criteria enabled the progressive diffusion of fatty acid monomers across the semipermeable proteinosome membrane, followed by in situ supramolecular assembly as the surfactant concentration increased to produce an ensemble of entrapped vesicles. As a consequence, antagonistic interactions in the parent host–guest protocells provided an internally driven pathway for self-reconfiguration into a nested protocell micro-architecture based on the spontaneous integration of fatty acid vesicles into single proteinosome microcapsules.

### Payload reorganisation in self-reconfigurable protocells

**Fig. 4:**
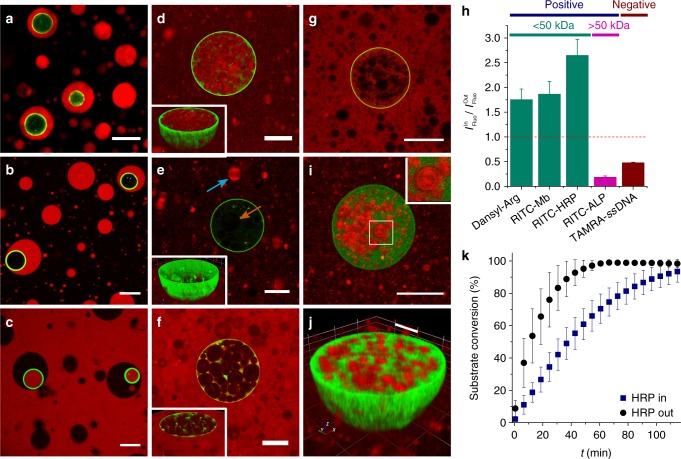
Payload reorganisation in self-reconfigurable host–guest protocells. **a**-**f** Confocal fluorescence microscopy images showing host–guest protocells consisting of GOx-containing FITC-labelled proteinosomes (green) entrapped within micelle coacervate droplets (unlabelled) prepared in the presence of red fluorescent RITC-HRP (**a**), RITC-ALP (**b**) or TAMRA-*ss*DNA (**c**), and after proteinosome-mediated disassembly of the host coacervate droplets (**d**–**f**, respectively). Initially, RITC-HRP and RITC-ALP are sequestered in the coacervate phase (**a**, **b**), whilst TAMRA-*ss*DNA is confined to the water-filled proteinosome interior and external solution (**c**). Enzyme-mediated disassembly gives rise to RITC-HRP transfer to the lumen of fatty acid vesicles housed within the proteinosome interior or present in the external solution (**d**), RITC-ALP transfer to vesicles formed outside the proteinosomes (**e**, blue arrow), but excluded from the proteinosome-internalised vesicles (**e**, orange arrow) and release of TAMRA-*ss*DNA to the external solution without transfer to the vesicles (**f**). Insets in **d**–**f** show 3D reconstructions of the proteinosomes. **g** Control experiments involving addition of RITC-HRP after completion of the micelle coacervate-to-vesicle transition showing no uptake of the solute by the preformed fatty acid vesicles. **h** Plot of normalised solute fluorescence intensity (*I*_IN_/*I*_OUT_) associated with vesicle-in-proteinosome hybrid protocells showing dependence of molecular weight and surface potential on payload uptake into the reconfigured nested protocells. Error bars represent the standard deviation of the ratio of fluorescence intensities. **i** Confocal fluorescence microscopy image of a single guest proteinosome prepared with a non-fluorescent membrane and containing FITC-GOx, and recorded after disassembly and transformation of the host–guest protocell. FITC-GOx molecules are excluded from the proteinosome-internalised Nile Red-stained fatty acid vesicles. Inset in **i** shows high-magnification image. **j** 3D reconstruction of a single guest proteinosome prepared with a non-fluorescent membrane and containing FITC-GOx, and recorded after disassembly and transformation of a host coacervate encapsulating RITC-HRP. RITC-HRP-containing vesicles (red) are housed within the FITC-GOx-containing (green) proteinosome. **k** Plots of time-dependent HRP-mediated conversion of Amplex Red into resorufin measured for vesicle/proteinosome guest–host protocells containing vesicle-entrapped HRP (blue), or control experiments in which HRP was added to the external phase of a vesicle suspension (black). Error bars represent the standard deviation of the substrate conversion (*n* = 3)

We exploited the self-induced reconfiguration of the enzymatically active proteinosome-in-coacervate droplets as a mechanism for spontaneously redistributing various molecular and macromolecular payloads between different compartments of the transforming nested microstructures. For this, we initially prepared hybrid protocells comprising different biomolecules specifically confined to the guest proteinosomes or host micelle coacervate droplet. Uptake of positively charged functionalized amino acids (α-dansyl-l-arginine), rhodamine B isothiocyanate (RITC)-labelled myoglobin (RITC-Mb, *M*_w_ = 17 kDa), RITC-HRP (*M*_w_ = 44 kDa) and RITC-labelled alkaline phosphatase (RITC-ALP, *M*_w_ ~150 kDa) into the coacervate phase was associated with high-equilibrium partition coefficients (*K* = 42 ± 2, 60 ± 3, 18 ± 1, and 6 ± 0.3, respectively, mean ± s.d., *n* = 3), and attributed to favourable electrostatic and/or hydrophobic interactions with the negatively charged fatty acid micelles^46,47^. As a consequence, these payloads were sequestered in the fatty acid micelle coacervate host droplets but not within the lumen of the guest proteinosomes (Fig. 4a, b). This spatial distribution was evident even though control experiments indicated that Mb and HRP were able to diffuse through the proteinosome membrane in the absence of the coacervate phase (Supplementary Fig. 12). In contrast, a low-molecular weight polyanionic carboxytetramethylrhodamine (TAMRA)-labelled single stranded oligonucleotide (TAMRA-ssDNA, 23 bases, *M*_w_ ~8.1 kDa, K = 0.1 ± 0.04, mean ± s.d., *n* = 3) did not partition into the negatively charged micelle coacervate host droplets, but could freely diffuse from the continuous aqueous phase to the interior of the guest proteinosomes (Fig. 4c).

Addition of 10 mM glucose to the host–guest protocells followed by enzyme-mediated acidification and disassembly of the micelle coacervate host resulted in transfer of α-dansyl-l-arginine, RITC-Mb and RITC-HRP from the coacervate phase into the aqueous interior of fatty acid vesicles produced in the external solution or within the released GOx-containing proteinosomes (Fig. 4d and Supplementary Fig. 13). In contrast, RITC-ALP in the coacervate droplets was trafficked only into vesicles present in the external environment due to the impermeability of the proteinosome membrane to this enzyme (Fig. 4e). On the other hand, TAMRA-ssDNA was released from the guest proteinosomes into the external solution after disassembly of the host protocell, and not transferred to the fatty acid vesicles (Fig. 4f). Control experiments in which the payloads were added to dispersions of preformed vesicles showed no evidence for uptake through the fatty acid membrane (Fig. 4g and Supplementary Fig. 13), indicating that in situ assembly of the vesicles from fatty acid monomers present within the proteinosome interior was responsible for solute encapsulation. The above observations were quantified by measuring the fluorescence intensities associated with the payloads present inside or outside the proteinosomes after GOx-mediated reconfiguration. For example, internalisation of the fatty acid vesicles within the proteinosomes resulted in a 1.7 to 2.6-fold accumulation of positively charged, low-molecular weight solutes (RITC-HRP, α-dansyl-l-arginine or RITC-labelled Mb) compared with the external continuous phase (Fig. 4h).

Self-reconfiguration of host–guest protocells containing spatially separated GOx and HRP produced multi-enzyme vesicle-in-proteinosome nested microstructures. Whereas GOx was retained within the aqueous phase of the released proteinosomes, HRP was entrapped specifically within the guest vesicles (Fig. 4i, j). As a consequence, the reconfigured nested protocells functioned synergistically when glucose and Amplex Red were added to the external solution to generate a spatially organised enzyme cascade (Fig. 4k). The initial reaction rate was 2.9 ± 1.3-fold (mean ± s.d., *n* = 3) slower compared to control experiments in which the HRP was present in the bulk solution of a dispersion of preformed vesicles. The difference was attributed to diffusional constraints associated with the requirement for substrate uptake through the proteinosome membrane and vesicle bilayer. Taken together, these results demonstrate that proteinosome-mediated disassembly of host micelle coacervate droplets into fatty acid vesicles is a mechanism to selectively transfer and accumulate payloads into membrane-bound nested hybrid protocells comprising multiple vesicle sub-compartments housed within single enzymatically active proteinosomes.

### Self-induced capture of proteinosomes

**Fig. 5:**
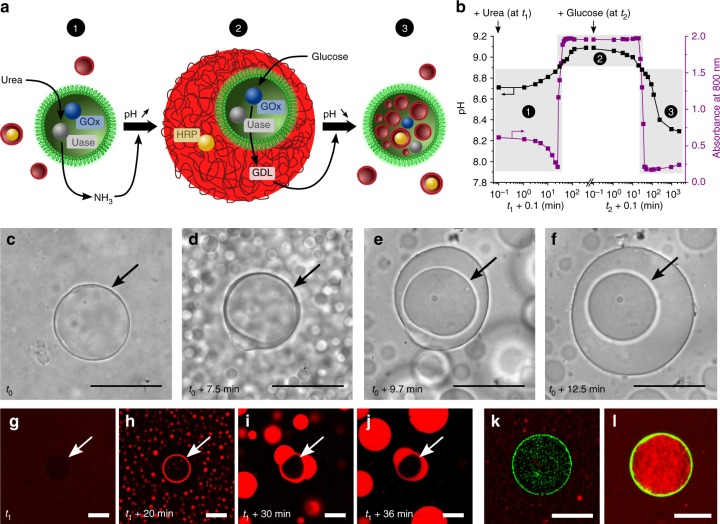
Self-induced capture of proteinosomes by enzyme-mediated vesicle-to-coacervate transitions. **a** Graphic showing restructuration of a mixed population of proteinosomes (green) containing encapsulated GOx (blue circle) and urease (Uase, grey circle) in the presence of non-encapsulated fatty acid vesicles (red) containing HRP (yellow circle) (state 1) into a nested proteinosome-in-coacervate micro-architecture (state 2) after addition of urea, followed by transformation into a vesicle-in-proteinosome structure (state 3) after addition of glucose. **b** Time-dependent plot showing sequential increase in pH after addition of 20 mM urea (at *t*_1_ = 0, black squares) to a mixed population of fatty acid vesicles and urease/GOx-containing proteinosomes, and associated rapid increase in absorbance (purple circles) due to the transformation of vesicles into micelle coacervate micro-droplets above pH 8.9. Subsequent addition of 10 mM glucose (at *t*_2_ = 0) produces a decrease in pH, and an associated rapid decrease in absorbance due to disassembly of the micelle coacervate droplets into vesicles below pH 8.9. The conversion from state 1 to 3 as labelled in **a** is highlighted. **c**–**f** Time series of optical microscopy images showing self-induced capture of a urease/GOx-containing proteinosome (arrow) via urease-mediated transformation of fatty acid vesicles into micelle coacervate droplets in the presence of urea (20 mM; time *t*_0_) (see Supplementary Movie 4). Scale bars, 50 μm. **g**–**j** As for **c**–**f**, but imaged by confocal fluorescence microscopy showing single proteinosome (white arrow) and vesicles (**g**) followed by formation of fatty acid coacervate droplets and initial stages of wetting of the urease/GOx-containing proteinosome 20 min after addition of urea (**h**). Capture of the proteinosome occurs via the interfacial coalescence of several coacervate droplets (**i**, **j**); scale bars, 50 μm. **k**, **l** Confocal fluorescence microscopy images of a single urease/GOx-containing FITC-labelled proteinosome (green fluorescence) in the presence of RITC-HRP-loaded fatty acid vesicles before (**k**) and after (**l**) sequential addition of urea (20 mM, 180 min) and glucose (10 mM, 240 min). Initially, vesicles containing RITC-HRP (red fluorescence) are excluded from the proteinosome lumen (ratio of red fluorescence inside vs. outside the proteinosomes, *I*_IN_/*I*_OUT_ = 0.5 ± 0.1), whilst after enzyme-mediated pH-induced host–guest protocell assembly and reconfiguration, many of the vesicles are located along with their payload in the proteinosome interior (*I*_IN_/*I*_OUT_ = 2.1 ± 0.1); scale bars, 50 μm

We sought to extend the functional importance of internally localised enzyme activity by augmenting the experimental design to include a initial step that involved self-induced capture of the proteinosomes via enzyme-mediated triggering of a vesicle-to-coacervate transformation (Fig. 5a). For this purpose, a population of proteinosomes containing both urease and GOx (Supplementary Fig. 14) was mixed with a dispersion of fatty acid vesicles at pH 8.7. Addition of urea at concentrations above or equal to 10 mM resulted in a gradual increase in pH over a period of ca. 180 min associated with the urease-mediated production of ammonia. As a consequence, the optical absorbance increased rapidly as the pH reached a value of 8.9 (Fig. 5b), indicating that the myristic acid vesicles were restructured into coacervate micelle micro-droplets. Subsequent addition of glucose (10 mM) produced a GOx-mediated pH decrease from ca. 9.1 to 8.3, which was associated with a marked decrease in the absorbance at pH 8.9 due to disassembly of the coacervate micelle droplets and re-establishment of the fatty acid vesicles. Control experiments performed in the absence or with lower concentrations of urea did not produce any absorbance change as the pH remained below the threshold value of 8.9 (Supplementary Fig. 15)

Optical microscopy images of the binary protocell population after exposure to urea (20 mM) confirmed that the fatty acid vesicles initially transformed into small coacervate micro-droplets (Fig. 5c, d). Significantly, continued urease activity inside the proteinosomes resulted in subsequent capture of the protein–polymer microcapsules by gradual coalescence of the coacervate micro-droplets around individual proteinosomes to produce proteinosome-in-coacervate nested protocells (Fig. 5e–j, Supplementary Movie 4). The host–guest protocells were then reconfigured by addition of glucose and triggering of GOx activity within the incarcerated proteinosomes (Fig. 5k, l). As a consequence, the vesicles and their payloads were transferred from the external environment into the lumen of the proteinosomes through a series of self-mediated protocell capture and reorganisation processes.

## Discussion

In this article, we describe the spontaneous assembly of proteinosome-in-coacervate nested protocells that can be dynamically restructured by internally generated enzyme reactions. We demonstrate synergistic (enzyme cascade) and antagonistic (guest-mediated host disassembly) modes of behaviour that are dependent on GOx activity within the incarcerated guest proteinosomes. In particular, we show how non-mutual interactions give rise to self-induced structural and functional reconfiguration of the host–guest arrangement along with selective transfer and accumulation of payloads to produce vesicles-in-proteinosome multi-compartmentalised protocells. Replacing the binary population of GOx-containing proteinosomes and HRP-containing micelle coacervate droplets with a mixture of urease/GOx-containing proteinosomes and HRP-encapsulated fatty acid vesicles gives rise to the self-induced capture of the proteinosomes and subsequent host–guest protocell restructuring via stepwise changes in pH associated with the interplay of the antagonistic enzymes.

From a general perspective, fabrication of micro-compartmentalised chemical systems with nested architectures and biomimetic properties is important for controlling the positional assembly of functional components^28,42^, programmed release of molecular payloads^28,33,42^ and spatiotemporal regulation of enzyme cascade reactions^32,35^. Use of these systems for modelling proto-organelle behaviour has focused predominately on nested structures comprising identical protocellular units rather than host–guest arrangements^30,31,34^ and have not addressed the advent of dynamical restructuring in hierarchical protocell constructs. In this regard, our results highlight opportunities for the fabrication of self-reconfigurable host–guest protocells and provide a first step towards the development of synthetic multi-compartmentalised cell-like models involving primitive aspects of spontaneous self-directed capture coupled to synergistic and antagonistic modes of interactivity.

## Methods

### Preparation of primary protocells

Myristic acid micelle coacervate micro-droplets were prepared by adding a twofold molar excess of guanidinium counterions to elongated myristate micelles at 32 °C (final pH ~ 9.5) (see Supplementary Methods). The synthesis of BSA–NH_2_/PNIPAAm nano-conjugates and their assembly into water-filled proteinosomes dispersed in aqueous solutions was undertaken as described in the Supplementary Methods. GOx, FITC- or RITC-labelled GOx, urease or RITC-labelled urease were encapsulated into the proteinosomes following a similar protocol.

### Spontaneous capture of proteinosomes in coacervate droplets

A binary population of interacting protocells was prepared by mixing an aqueous dispersion of GOx- or FITC-GOx-containing BSA–NH_2_/PNIPAAm proteinosomes with a freshly prepared myristic acid micelle coacervate droplet suspension at pH ~9.5 and 32 °C. Typically, the primary protocells were mixed in an Eppendorf tube, which was tapped gently to mix the two populations. The final proteinosome : coacervate droplet number density ratio was varied from 1:140, 1:26, 1:12 to 1:7 by adding 2, 10, 20 or 30 μL of the proteinosome suspension to a micelle coacervate dispersion to reach a final volume of 100 μL. Typically, covalent attachment of FITC was used to label the proteinosome membrane, and Nile Red used as a stain for the micelle coacervate droplets. The resulting proteinosome-in-coacervate host–guest protocells were loaded between a glass slide and coverslip, and mounted on a microscope sample holder. Uptake and confinement of the proteinosomes within the fatty acid micelle coacervate droplets was assessed by confocal fluorescence microscopy and FACS statistical analysis (20,000 particles per run). The statistical distributions and equilibrium partition coefficient associated with proteinosome capture within the micelle coacervate droplets under different conditions were derived from image processing with ImageJ. See Supplementary Methods for more details.

Spontaneous sequestration of individual proteinosomes within the fatty acid micelle coacervate droplets was monitored in real time using a Leica DMI3000 epifluorescence microscope equipped with a 20× objective lens and a Hg-Arc lamp light source combined with appropriate filter cubes to select the excited and emitted fluorescence wavelength range.

### Spatially coupled enzyme cascade reactions

Fatty acid micelle coacervate droplets loaded with 200 nM HRP and 1 mM oPD were prepared by adding aliquots of stock solutions of HRP and *o*PD (10 μM and 50 mM, respectively) to a coacervate droplet suspension prepared at 32 °C and pH ~9.5. The HRP-containing coacervate droplets were mixed with a population of GOx-containing proteinosomes (proteinosome : coacervate droplet number density ratio = 1:12) to produce a suspension of bi-enzyme-containing proteinosome-in-coacervate host–guest protocells. For experiments involving the spatially segregated protocells, 350 μL of GOx-containing proteinosomes were added to a 0.5 mL dialysis device (3500 Da, MWCO, Slide-A-Lyzer MINI, ThermoFisher Scientific) that was placed on top of an eppendorf tube filled with 1.4 mL of a micelle coacervate droplet suspension loaded with HRP (200 nM) and *o*PD (1 mM). In this way, the two protocell solutions were in different reservoirs and in contact only through the dialysis membrane, namely only small molecules could diffuse from one protocell population to the other. The enzyme cascade reaction was initiated by addition of glucose (1 mM) to the suspension of host–guest protocells or to the proteinosome suspension present in the spatially segregated system. Changes in fluorescence associated with the HRP-mediated oxidation of *o*PD into DAP in the presence of GOx-produced H_2_O_2_ was monitored overtime (CLARIOstar plate reader (BMG LabTech)) by loading 50 μL aliquots of the host–guest protocell suspension or the micelle coacervate droplet suspension for the spatially separated system into a 96 μL well plate (*λ*_ex_ = 410 ± 16 nm, *λ*_em_ = 550 ± 20 nm). For experiments involving the spatially segregated protocells, 10 μL of the proteinosome suspension were discarded from the dialysis device after withdrawal of each aliquot of the micelle coacervate suspension to maintain a number density ratio of 1:12. Control experiments were performed in the absence of glucose. The initial rate of reaction was extracted in each case by fitting the initial (*t* < 20 min) fluorescence changes to a line. Epifluorescence microscopy imaging was also used to monitor the cascade reaction in the presence or absence of 1 mM glucose.

### Disassembly of coacervate droplets by captured proteinosomes

A given volume fraction of proteinosomes containing 10.0 mg mL^−1^ of GOx was added to a freshly made myristic acid micelle coacervate suspension at 32 °C and pH ~10.0 to give a final proteinosome : coacervate number density ratio between 1:7 and 1:140. The mixture was homogenised by gentle pipetting, then kept at 32 °C under continuous magnetic stirring to produce a suspension of coacervate droplets containing captured proteinosomes. The GOx enzyme reaction was initiated by addition of a given volume of a 1 M aqueous stock solution of glucose (*t* = 0) to give a final glucose concentration of 5–20 mM, and the pH monitored overtime (Supplementary Methods).

Enzymatically induced proteinosome-mediated disassembly of the fatty acid micelle host coacervate droplets was monitored by bright-field and epifluorescence imaging of the protocell populations every 10 or 30 s. Alternatively, the proteinosome-mediated disassembly of Nile Red-doped host micelle coacervate droplets was monitored by recording confocal microscopy images every 30 s by maintaining the FITC-labelled proteinosomes in the focal plane. Self-assembly of fatty acid vesicles-in-proteinosomes during disassembly of the host droplets, as well as in various control experiments, was monitored by confocal fluorescence microscopy (Supplementary Methods).

The proteinosome-mediated micelle coacervate disassembly process was further analysed statistically by time-dependent FACS measurements (Supplementary Methods).

### Coacervate droplet to vesicle molecular transfer

Myristic acid micelle coacervate micro-droplets were loaded with different fluorescently labelled solutes (α-dansyl-l-arginine, RITC-Mb, RITC-HRP, RITC-ALP or TAMRA-*ss*DNA) by adding aliquots of stock aqueous solutions of the solutes to the droplet suspension at pH ~9.5 and 32 °C (Supplementary Methods). Solute sequestration in the coacervate droplets was assessed by confocal microscopy imaging, as well as by measurement of partition coefficients (Supplementary Methods). An aqueous suspension of GOx-loaded FITC-tagged proteinosomes was added to the micelle coacervate dispersion (proteinosome : coacervate number density ratio, 1:12), followed by addition of glucose (final concentration, 10 mM). The mixture was kept at 32 °C under gentle magnetic stirring for 4 h, then loaded between a glass slide and coverslip for confocal or epifluorescence microscopy imaging. Control experiments were performed by adding the solutes after the proteinosome-mediated disassembly of the non-loaded micelle coacervate micro-droplets and transformation into fatty acid vesicles.

The enzymatic activity of the transferred RITC-HRP was determined using standard procedures (Supplementary Methods).

### Self-induced capture of proteinosomes

A 100 μL aliquot of proteinosomes containing 15.0 mg mL^−1^ of urease and 5.0 mg mL^−1^ of GOx was added to 400 μL of a freshly made myristic acid vesicle suspension at 32 °C and pH ~8.7. The mixture was homogenised by gentle pipetting, and then the pH was carefully re-adjusted to 8.7 by using 0.1 M HCl or NaOH, and the solution kept at 32 °C under continuous magnetic stirring. The urease-mediated enzyme reaction was initiated by addition of a given volume of a 1 M aqueous stock solution of urea (*t*_1_ = 0) to give a final urea concentration of 20 mM, and the pH and absorbance monitored overtime. After ~5 h, the pH reached a plateau value, and a given volume of a 1 M aqueous stock solution of glucose (*t*_2_ = 0) was then added to give a final glucose concentration of 10 mM. Subsequent changes in pH and absorbance of the solution were monitored overtime.

Urease-induced proteinosome-mediated assembly of Nile Red-doped host micelle coacervate droplets was monitored every 10 or 30 s by acquiring images of the protocell populations mounted on a sealed capillary slide using bright-field and confocal fluorescence microscopies. Transfer of RITC-HRP from vesicles initially present in the external environment to vesicles entrapped within the proteinosome lumen was established by imaging the urease/GOx-containing FITC-labelled proteinosomes by confocal fluorescence microscopy before and after sequential addition of urea (20 mM, 180 min) and glucose (10 mM, 240 min).

## Electronic supplementary material

Supplementary Information

Description of Additional Supplementary Files

Supplementary Movie 1

Supplementary Movie 2

Supplementary Movie 3

Supplementary Movie 4

## Data Availability

The authors declare that all relevant data supporting the finding of this study are available within the paper and its Supplementary information files. Additional data are available from the corresponding author upon request.
