# Supplementary material for: Antagonistic chemical coupling in self-reconfigurable host–guest protocells
Source: Nat Commun. 2018 Sep 7;9:3652. doi: 10.1038/s41467-018-06087-3 (PMC6128866; doi:10.1038/s41467-018-06087-3)
Supplement: Supplementary file 1 — Supplementary Information [file 41467_2018_6087_MOESM1_ESM.pdf]

Supplementary Information

Antagonistic chemical coupling in self-reconfigurable host-guest protocells

Martin *et al.*

Supplementary Figures

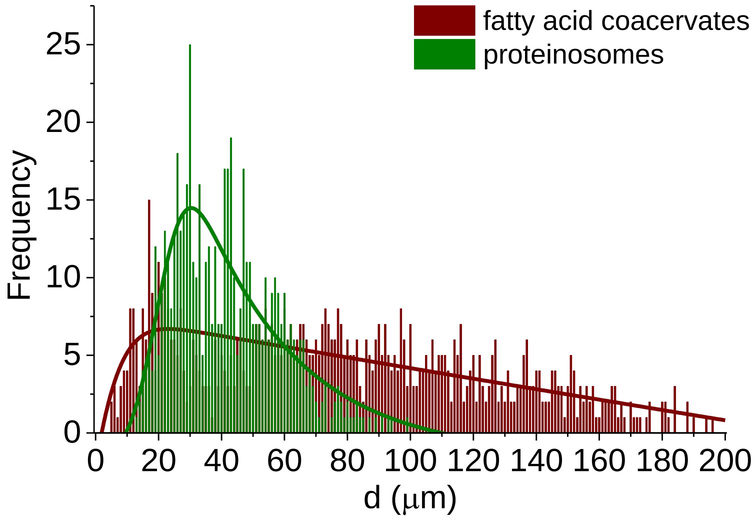

Supplementary Figure 1. Size distribution of protocells. Distribution in the diameter, *d*, of Nile red-doped fatty acid micelle coacervate micro-droplets (red) and FITC-labelled GOx-containing BSA-NH_2/PNIPAAm proteinosomes (green) measured from epifluorescence microscopy images of the individual protocell populations. The size distributions were fitted using exponentially modified Gaussian peak functions (shown as solid lines).

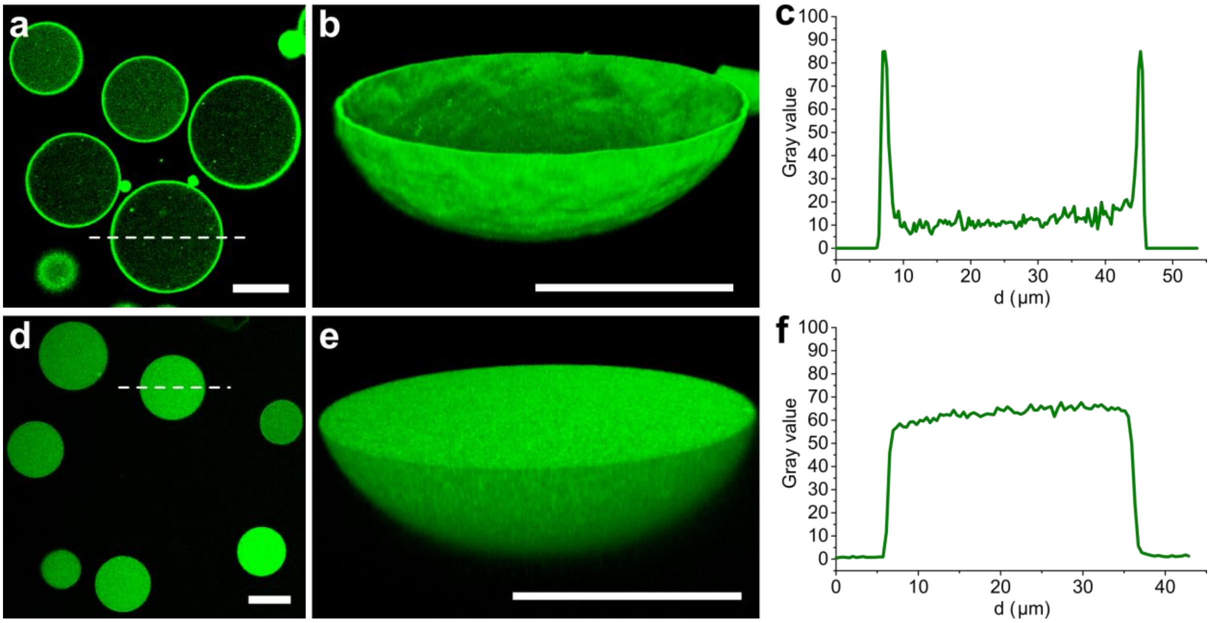

Supplementary Figure 2. Imaging of GOx-containing proteinosomes. a,**d**, Confocal fluorescence microscopy images of (**a**) FITC-membrane labelled proteinosomes containing GOx, or (**d**) non-fluorescently labelled proteinosomes with encapsulated FITC-tagged GOx, showing the presence of a BSA-PNIPAAm nanoconjugate-based membrane enclosing a water-filled lumen filled with a homogeneous distribution of encapsulated enzyme molecules; scale bars, 20 µm. **b**,**e**, Corresponding 3D reconstructions of a single proteinosome prepared as in (**a**) and (**d**), respectively; scale bars, 20 µm. **c**,**f**, Corresponding fluorescence intensity profiles across a single proteinosome (dotted lines in **a** and **d**, respectively).

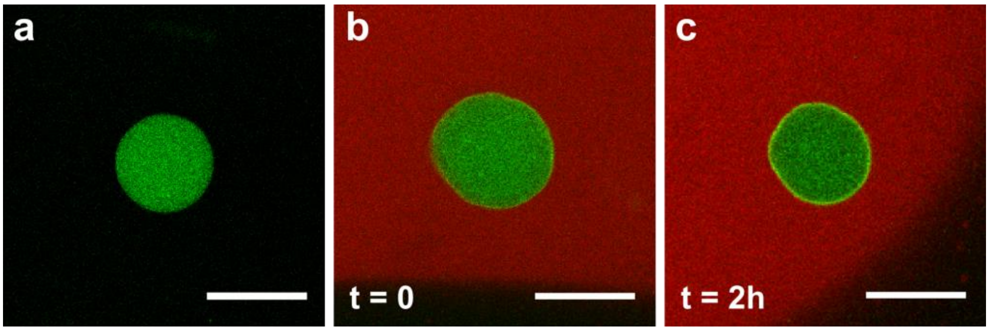

Supplementary Figure 3. Retention of FITC-GOx within coacervate-captured proteinosomes. Confocal fluorescence microscopy images of unlabelled proteinosomes containing FITC-GOx recorded (**a**) in water, or (**b**,**c**) inside a Nile Red-doped myristic acid micelle coacervate micro-droplets (**b**) immediately after mixing the two populations or (**c**) after 2 hours of incubation. Images **b** and **c** show the same proteinosome at t = 0 and at t = 2h; scale bar, 50 µm.

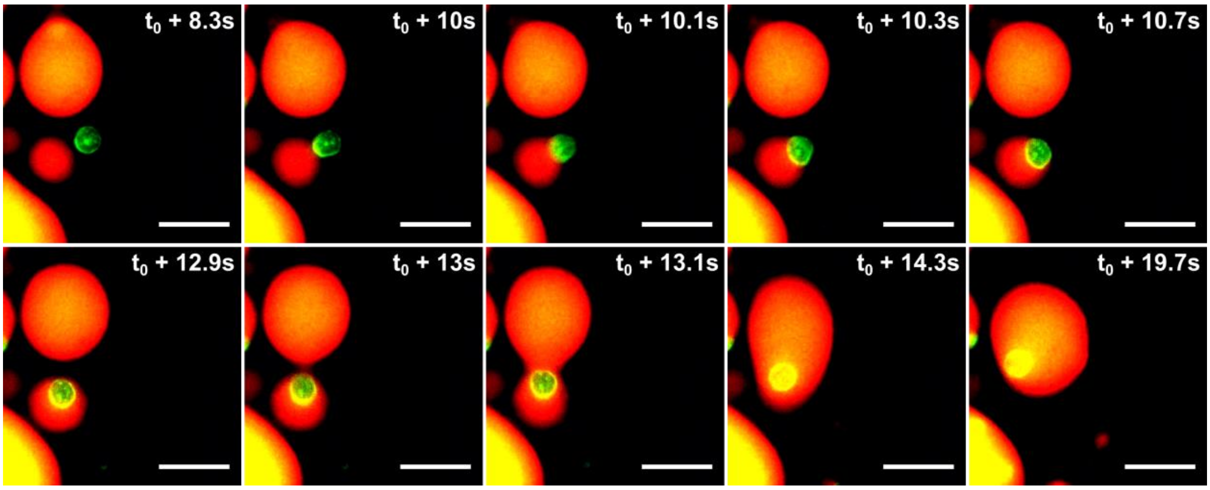

Supplementary Figure 4. Spontaneous capture of proteinosomes by fatty acid micelle coacervate micro-droplets. Snapshots from Supplementary Movie 1 showing wetting and capture of an individual proteinosome labelled with FITC (green) by a large single micelle coacervate droplet doped with Nile Red (red), followed by coalescence with a larger droplet; scale bars, 50 µm.

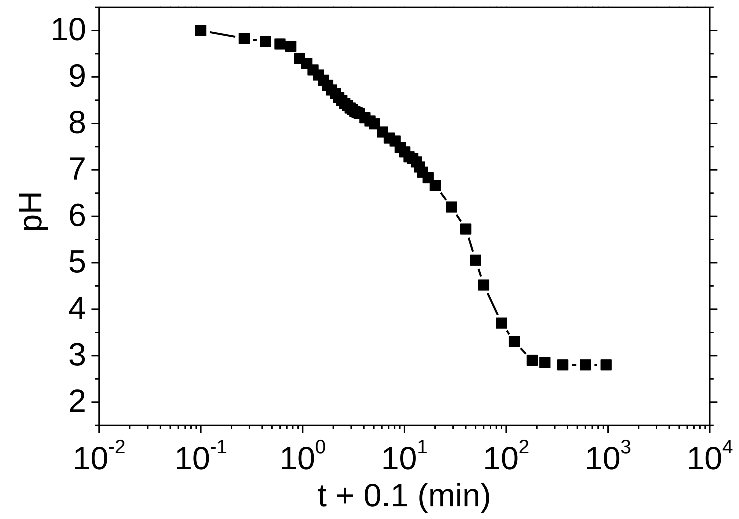

Supplementary Figure 5. Proteinosome-mediated pH changes in water. Plot showing time-dependent decrease in pH in an aqueous suspension of GOx-containing proteinosomes after addition of 10 mM glucose. The pH was initially adjusted to 10.0 with NaOH (0.1M).

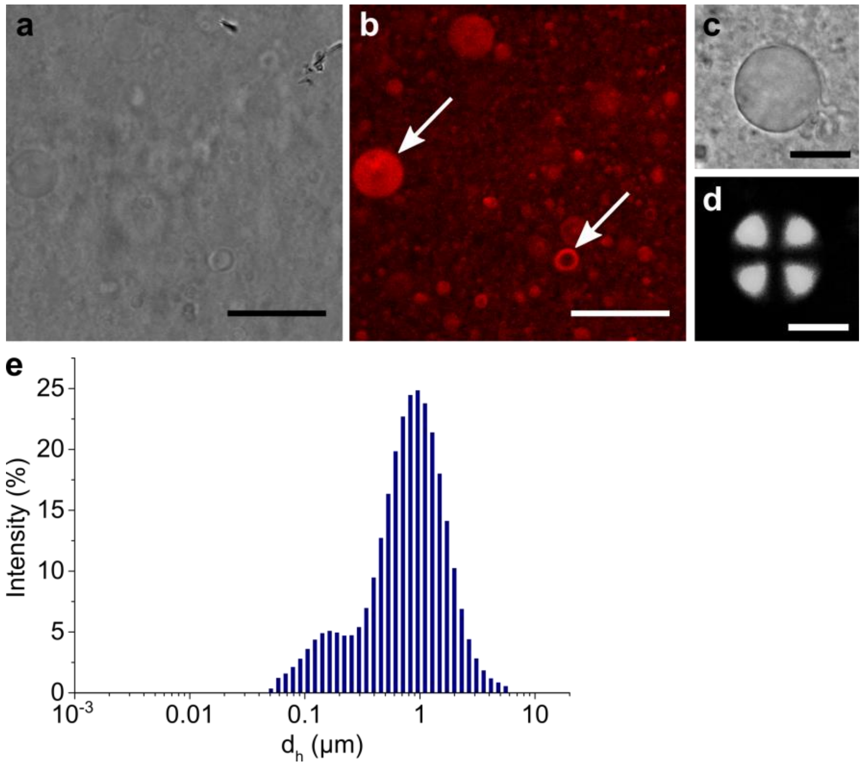

Supplementary Figure 6. Characterization of fatty acid vesicles. **a**,**b**, Optical (**a**) and confocal fluorescence (**b**) microscopy images of myristic acid vesicles (white arrows) formed after proteinosome-induced disassembly of Nile Red-doped micelle coacervate droplets (final pH, ~8.0); scale bars, 20 µm. **c**,**d**, Single fatty acid vesicle viewed by optical microscopy (**c**) under aligned or (**d**) crossed polarisers showing Maltese cross pattern consistent with a multilamellar structure; scale bars, 10 µm. **e**, Distribution of the hydrodynamic diameter, d_h, of fatty acid vesicles formed after proteinosome-induced micelle coacervate micro-droplet disassembly. Data from dynamic light scattering.

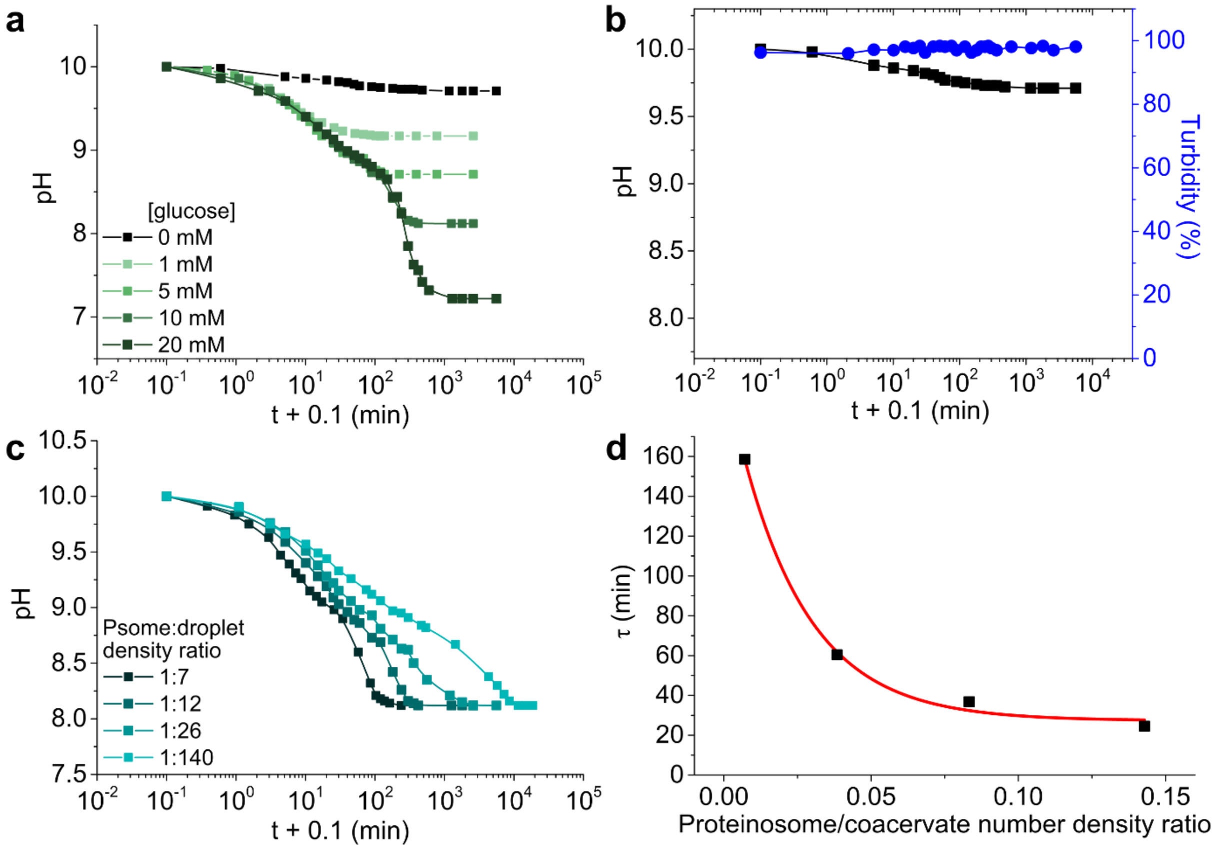

Supplementary Figure 7. Proteinosome-mediated pH decrease in fatty acid micelle coacervate suspensions. a, Time-dependent plots showing changes in pH in an aqueous dispersion of host-guest protocells comprising GOx-containing proteinosomes entrapped within myristic acid micelle coacervate micro-droplets at a fixed number density ratio (1:12) and varying initial glucose concentrations. **b**, Plot as in (**a**) but in the absence of glucose showing minimal change in pH (black) and turbidity (blue) over ~20h. **c**, Plot as in (**a**) but at varying number density ratios and fixed initial glucose concentration (10 mM). **d**, Plot of the characteristic time τ required to reach a threshold pH value of 8.9 as a function of the proteinosome : coacervate number density ratio. In general, buffering of the solution by myristic acid resulted in slower decreases in pH and higher final pH values compared with control experiments undertaken on GOx-containing proteinosomes dispersed in pure water (see Supplementary Figure 5). The data was fitted to a mono-exponential function (shown in red) not a hyperbola as expected for a simple one-step enzyme reaction. We attribute the increased complexity to; (i) production of glucono-δ-lactone (GDL) by GOx, (ii) spontaneous hydrolysis of GDL into gluconic acid and (iii) the counter-acting buffering effect from myristic acid (protonation/deprotonation). In addition, GOx activity and the rate of GDL hydrolysis depend on pH and will therefore change as the pH decreases with time.

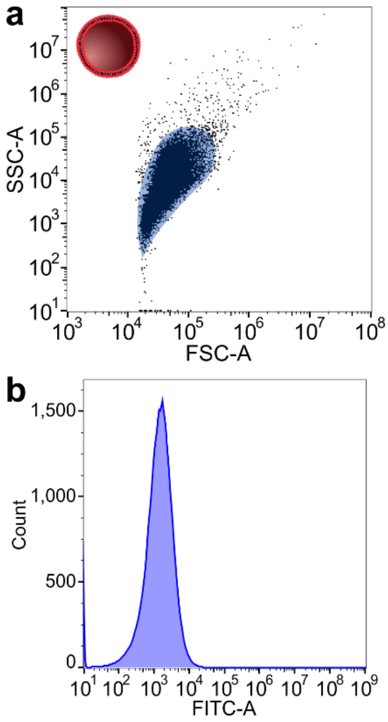

Supplementary Figure 8. FACS analysis of fatty acid vesicles. a, 2D dot plots of side-scattered light (FSC) versus forward-scattered light (SSC) for a control sample of myristic acid vesicles formed by addition of HCl (1M) to a micelle coacervate droplet dispersion (final pH ~8.5). **b**, Corresponding histogram of green fluorescent signal (FITC-A); the low fluorescence level (~10^3) corresponds to background noise.

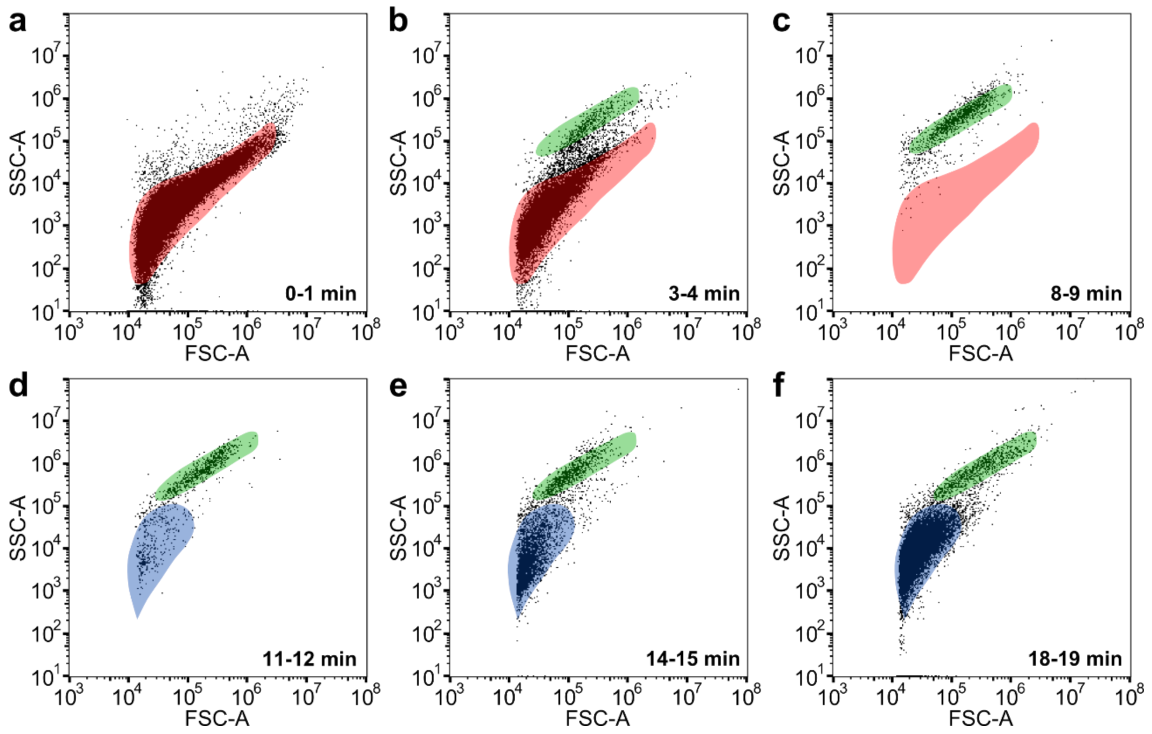

Supplementary Figure 9. FACS studies of proteinosome-mediated coacervate droplet disassembly and reconfiguration into vesicles. 2D dot plots of side-scattered light (SSC) versus forward-scattered light (FSC) for host-guest protocells at an initial pH of 9.1 comprising GOx-containing proteinosomes entrapped within unlabelled myristic acid micelle coacervate droplets (**a**) 0-1 min, (**b**) 3-4 min, (**c**) 8-9 min, (**d**) 11-12 min, (**e**) 14-15 min and (**f**) 18-19 min after addition of 10 mM glucose (total number of particles = 20,000; proteinosome : coacervate droplet number density ratio = 1:12). The time series of FACS plots shows the progressive disappearance of the fatty acid micelle coacervate population (red domain), release of the proteinosomes (green domain), followed by the gradual formation of vesicles (blue domain).

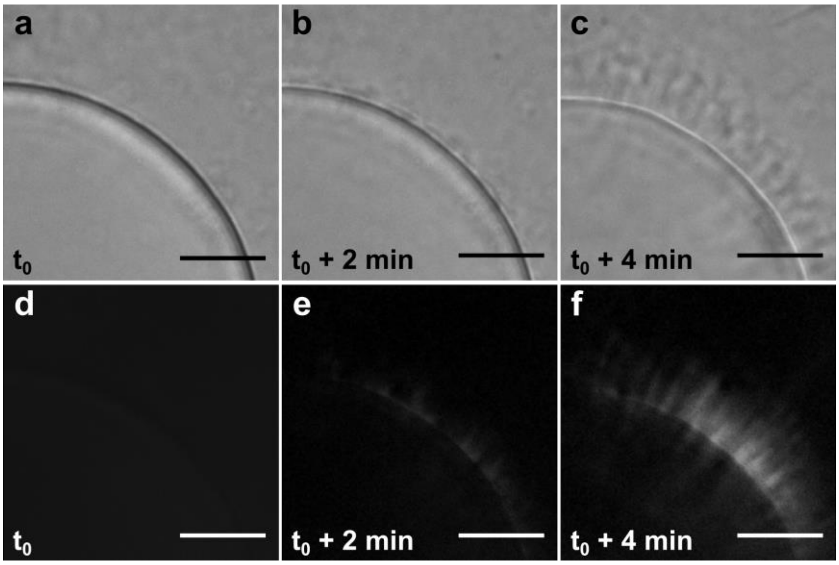

Supplementary Figure 10. Birefringence characterization of a disassembling coacervate droplet. a-c, Bright-field optical microscopy images recorded on the edge of a myristic acid micelle coacervate droplet during proteinosome guest-mediated disassembly of the coacervate host, showing the progressive formation of low optical contrast structures at the coacervate/water interface; scale bars, 20 µm. **d**-**f**, Corresponding images recorded under crossed polarisers that show a marked birefringent signature from the protrusions formed during micelle coacervate disassembly; scale bars, 20 µm.

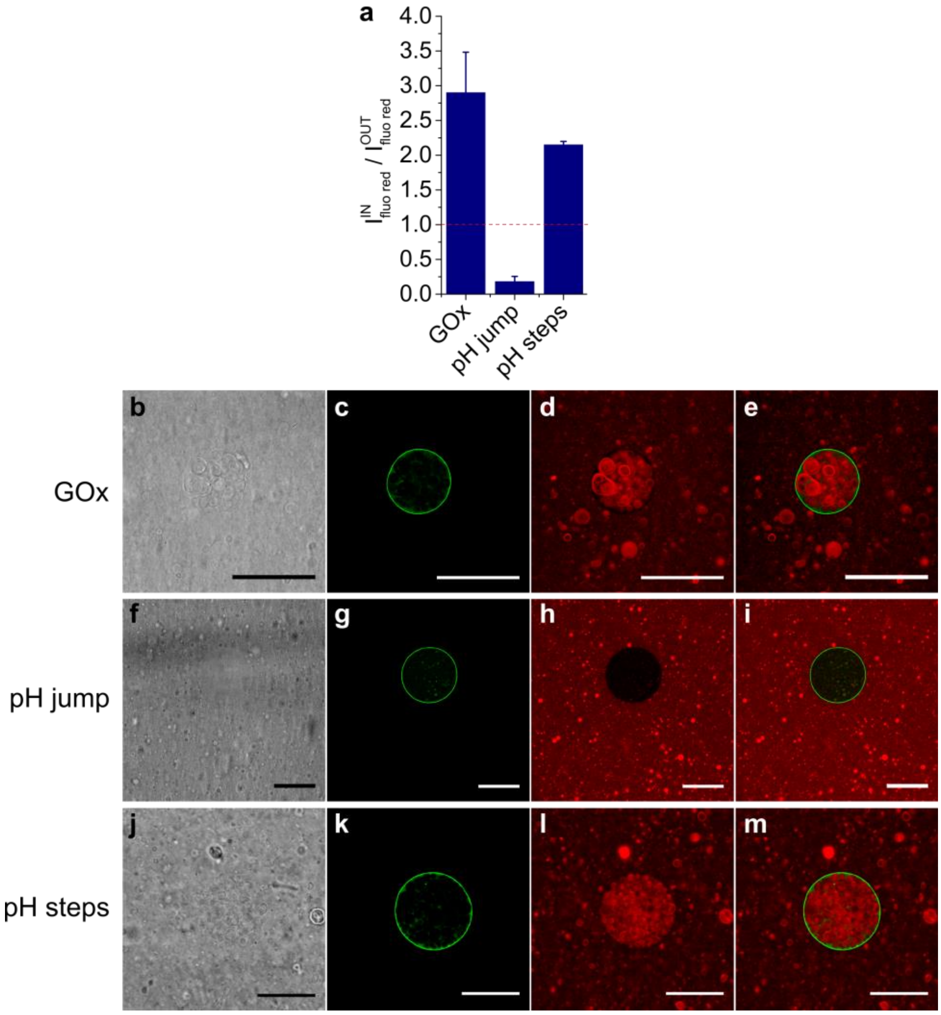

Supplementary Figure 11. Spatial localisation of fatty acid vesicles after disassembly of the micelle coacervate droplets by different procedures. **a**, Red fluorescence intensity per unit area recorded inside FITC-labelled GOx-containing proteinosomes and normalized to the background fluorescence intensity outside the proteinosomes after different procedures were used to disassemble the host coacervate protocells by acidification. Red fluorescence is associated with formation of Nile Red-doped fatty acid vesicles: GOx, proteinosome-mediated micelle coacervate disassembly after enzyme reaction with 10 mM glucose for 4h; pH jump, one-step pH decrease by addition of 1 M HCl to a dispersion of the host-guest protocells to reach a final pH of ~8.5; pH steps, stepwise pH decrease by addition of aliquots of HCl (0.02M) to a dispersion of the host-guest protocells to reach a final pH of ~8.5. Error bars represent the standard deviation of the ratio of fluorescence intensities. **b-m**, Corresponding optical (**b**,**f**,**j**) and confocal fluorescence (**c**-**e**, **g**-**i**, **k**-**m**) microscopy images of a single proteinosome imaged in the green channel, red channel, and overlaid fluorescence signals for experiments referred to in **a**; (**b**-**e**) GOx, (**f**-**i**) pH jump and (**j**-**m**) pH steps, respectively; scale bars, 20 µm.

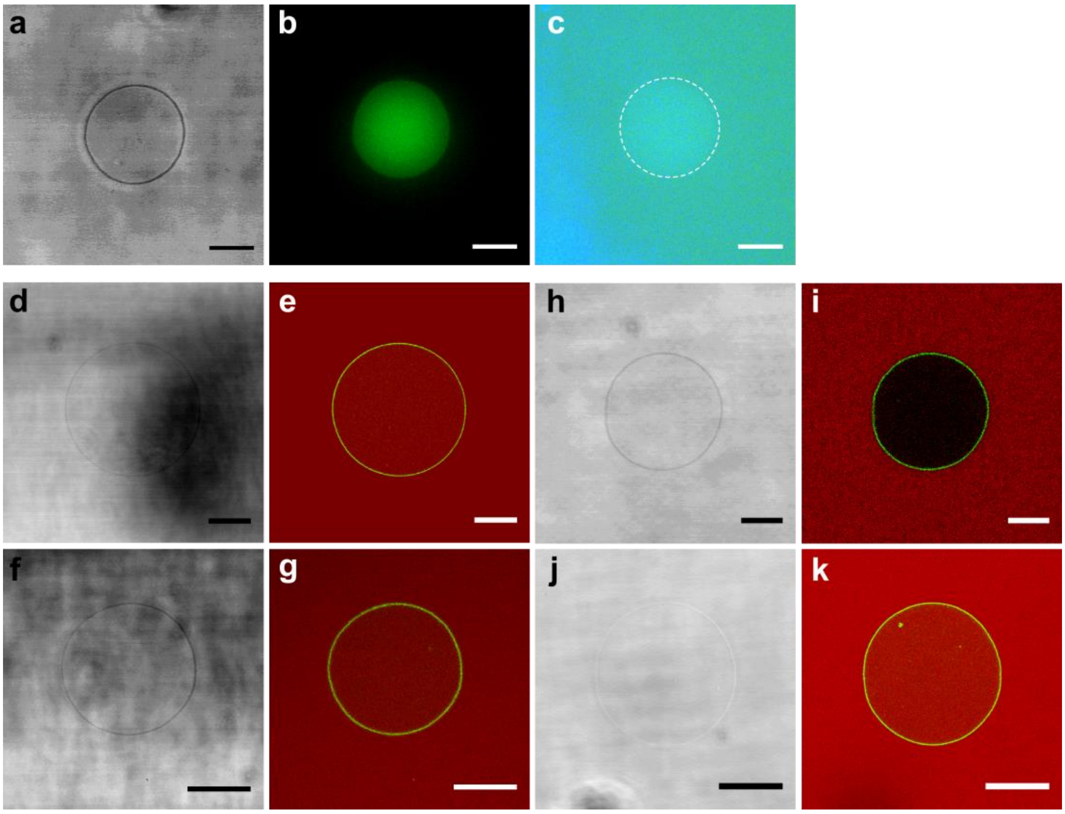

Supplementary Figure 12. Permeability studies of proteinosomes. a-**c**, Optical (**a**) and epifluorescence (**b**,**c**) microscopy images of a single FITC-labelled GOx-containing proteinosome (green channel) after addition of α-dansyl-L-arginine (cyan channel) to the external solution, showing passive diffusion of the molecular solute across the semi-permeable proteinosome membrane. White dotted lines highlight the positions of the proteinosome. **d**-**k**, Optical (**d**,**f**,**h**,**j**) and confocal fluorescence (**e**,**g**,**i**,**k**) microscopy images of single FITC-proteinosomes after addition to the external solution of (**d**,**e**) RITC-Mb, (**f**,**g**) RITC-HRP, (**h**,**i**) RITC-ALP and (**j**,**k**) TAMRA-*ss*DNA, showing (**d**-**g**,**j**,**k**) diffusion of solutes with a molecular weight < 50 kDa and (**h**,**i**) exclusion of solutes with a molecular weight > 50 kDa across the proteinosome membrane. Scale bars, 20 μm.

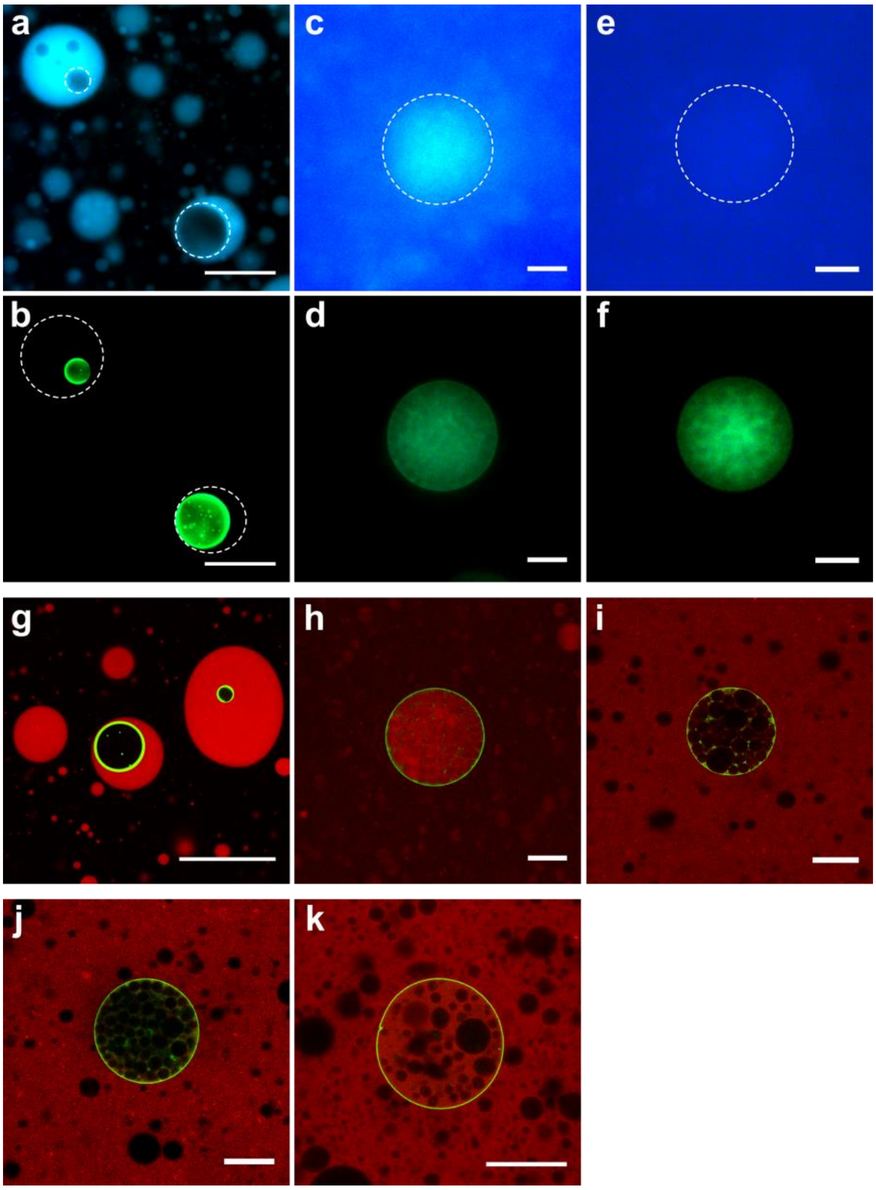

Supplementary Figure 13. Payload trafficking during host-guest protocells reconfiguration. a-**f**, Epifluorescence microscopy images of host-guest protocells consisting of FITC-labelled GOx-containing guest proteinosome (green channel) entrapped within single micelle coacervate droplets in the presence of α-dansyl-L-arginine (cyan channel) (**a**,**b**) before and (**c**,**d**) after proteinosome-mediated disassembly and transformation of the myristic acid coacervate to fatty acid vesicles. Initially, the functionalized amino acid resides preferentially in the micelle coacervate but is transferred to the guest proteinosomes after disassembly of the droplets. Control experiments in which the amino acid was added after the coacervate-to-vesicle transition was completed showed no accumulation of α-dansyl-L-arginine in the proteinosomes (**e**,**f**). White dotted lines highlight the positions of the coacervate droplets or proteinosomes. **g**,**h**, Confocal fluorescence microscopy images of samples prepared and processed as in (**a**,**c**) but in the presence of RITC-labelled Mb (**g**) before and (**h**) after proteinosome-mediated pH decrease, showing transfer and accumulation of RITC-Mb in the proteinosome-internalized fatty acid vesicles. **i,** Control experiments in which RITC-Mb was added after the coacervate-to-vesicle transition was completed showed no accumulation of the protein in the fatty acid vesicles. Scale bars, 50 μm (**a**,**b**,**g**) or 20 μm (**c**-**f**,**h**,**i**). **j**,**k**, Confocal fluorescence microscopy images for control experiments involving addition of (**j**) RITC-ALP or (**k**) TAMRA-*ss*DNA after completion of the micelle coacervate-to-vesicle transition showing no uptake of the solutes by the preformed fatty acid vesicles; scale bars, 20 μm.

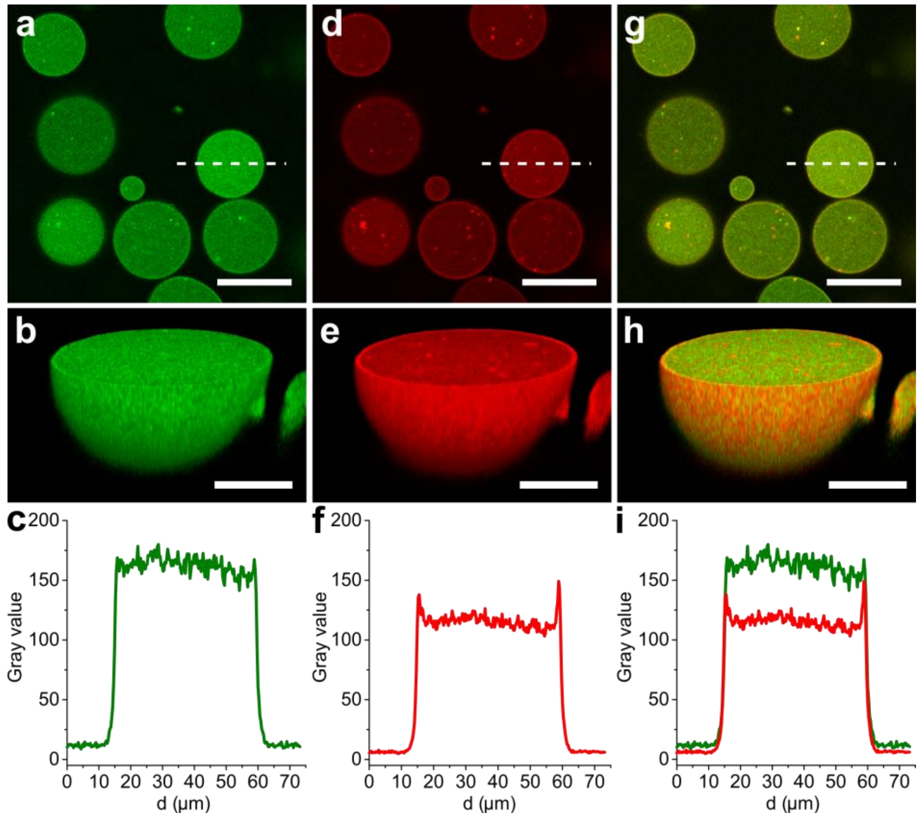

Supplementary Figure 14. Encapsulation of urease and glucose oxidase in proteinosomes. a,**d**,**g**, Confocal fluorescence microscopy images of non-fluorescently labelled proteinosomes with (**a**) encapsulated FITC-tagged urease and (**d**) RITC-labelled GOx and (**g**) overlaid fluorescence signals showing the homogeneous distribution of encapsulated enzyme molecules; scale bars, 50 µm. **b**,**e**,**h**, Corresponding 3D reconstructions of a single proteinosome; scale bars, 20 µm. **c**,**f**,**I**, Corresponding fluorescence intensity profiles across a single proteinosome (dotted lines in **a**, **d** and **g** respectively).

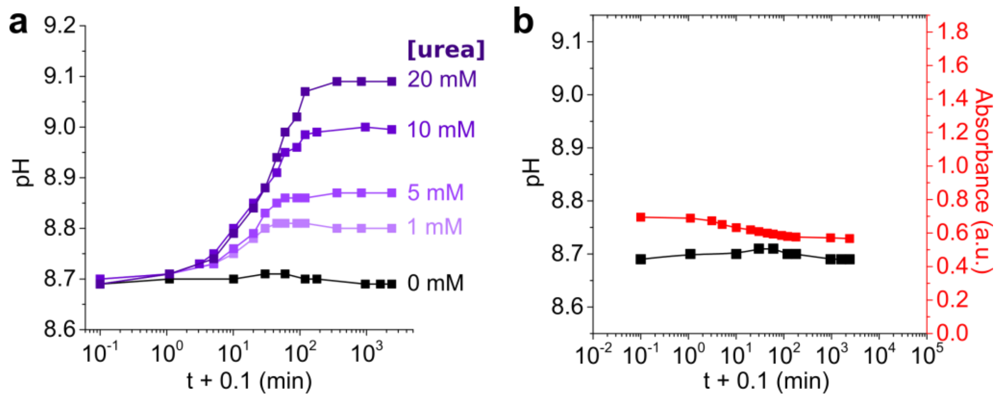

Supplementary Figure 15. Proteinosomes-mediated pH increase in fatty acid micelle coacervate suspensions. a, Time-dependent plots showing changes in pH in an aqueous dispersion of urease- and GOx-containing proteinosomes mixed with myristic acid vesicles and varying initial urea concentrations. **b**, Plot as in (**a**) but in the absence of urea showing minimal change in pH (black) and absorbance (red) over ~20h.

Supplementary Methods

Materials: The following chemicals were purchased from Sigma-Aldrich and used as received: myristic acid (MA, ≥99%), guanidinium hydrochloride (Gu, ≥99%), sodium hydroxide (97%), Triton X-114, fluorescein isothiocyanate (FITC, ~98% HPLC) , rhodamine B isothiocyanate (RITC, mixed isomers, BioReagent), 1,6-hexanediamine (HMDA, 98%), bovine serum albumin (BSA, heat shock fraction, > 98%), N-(3-dimethylaminopropyl)-N'-ethylcarbodiimide hydrochloride (EDC), hydrogen chloride (HCl, 35%) 2-ethyl-1-hexanol (>99.6%), O,O$^{/}$-bis[2-(N-succinimidyl-succinylamino) ethyl] polyethylene glycol (PEG-bis-(N-succinimidyl succinate), M_w = 2,000 g mol^{-1}), glucose oxidase (GOx, from *Apsergillus Niger*), α-D-glucose (96%), horseradish peroxidase (HRP, lyophilized), *ortho*-phenylenediamine (*o*PD, 99.5%), Amplex Red (>98% HPLC), alkaline phosphatase (ALP, from bovine intestinal mucosa, BioUltra), myoglobin (Mb, from equine skeletal muscle), α-dansyl-L-arginine hydrochloride. Single-stranded oligonucleotide with 56-TAMRA modification (TAMRA-*ss*DNA, 23 bases) was purchased from Integrated DNA Technologies Inc., Belgium, as a HPLC-purified freeze-dried solid, and dissolved in DNAse-free water to prepare a 100 µM stock solution.

Preparation of fatty acid micelle coacervate micro-droplets: Myristic acid micelle coacervate micro-droplets were prepared following a previously reported procedure.[1,2] Briefly, a stock solution of guanidinium-decorated myristate micelles was prepared by dispersing 5 g of myristic acid (MA) in 456 mL of Milli-Q water, followed by the addition of 22 mL of NaOH 1M and 22 mL of a 1M solution of guanidinium (Gu) hydrochloride to reach a final MA/NaOH/Gu molar ratio of 1:1:1. The solution was heated at 65°C and vigorously shaken to ensure the complete dissolution of fatty acid. Micelle coacervate micro-droplets suspensions were prepared by adding an excess of GuHCl to reach a final MA/NaOH/Gu molar ratio of ~1:1:2. Typically, 300 µL of a 1M aqueous solution of GuHCl were added to 5 mL of 1:1:1 mol/mol/mol MA/NaOH/Gu stock solution at 65°C, then shaken and stored at 35°C. 200 µL of Triton X-114 at 75 mg mL^{-1} were further added to stabilize the droplets at lower temperature.

Synthesis of FITC-labelled BSA-NH_2/PNIPAAm nanoconjugates: Amine-modified bovine serum albumin (BSA-NH_2) was synthesized according to a previously described method.[3] Briefly, 1,6-hexanediamine (HMDA) was covalently conjugated to aspartic and glutamic acid residues on the surface of BSA via carbodiimide activation. Typically, an aqueous solution of HDMA (500 mg, 4.3 mmol, 5 mL) was adjusted to pH 6.5 using 5M HCl and added dropwise to a stirred solution of BSA (100 mg, 1.5 µmol, 5 mL). The pH was readjusted if necessary to 6.5 with 0.1M HCl. The coupling reaction was initiated by adding 50 mg N-(3-dimethylaminopropyl)-N'-ethylcarbodiimide hydrochloride (EDC) to the stirred solution. The pH value was maintained at 6.5 by adding aliquots of 0.1M HCl, and another 50 mg of EDC were added after 4h. The solution was then stirred overnight, then filtered (Sartorius Ministart® NML syringe filters, 45 µm) to remove any precipitate. The BSA-NH_2-containing supernatant was then dialyzed (Medicell, dialyzing tubing, MWCO 12-14 kDa) for 2 days against Milli-Q water, and freeze-dried.

Labelling of BSA-NH_2 with fluorescein isothiocyanate (FITC) was undertaken according to the following procedure: 100 µL of a 2 mg mL^{-1} FITC solution in DMSO were added dropwise to BSA-NH_2 (20 mg) dissolved in 10 mL carbonate buffer (0.1M, pH 9.0). The mixture was stirred at room temperature for 5 hours, then dialyzed (Medicell, dialyzing tubing, MWCO 12-14 kDa) for 2 days against Milli-Q water, and freeze-dried. Based on UV-vis spectroscopy measurements, there were on average 1-2 dye molecules per protein.

End-capped mercaptothiazoline-activated PNIPAAm (M_n = 8,800 g mol^{-1}) was synthesized following a previously reported procedure.[3] An aqueous solution of the polymer (10 mg in 5 mL) was added dropwise to a stirred solution of FITC-labelled BSA-NH_2 (10 mg in 5 mL phosphate buffer 0.1M, pH = 8.0). The mixed solution was stirred for 12h at 4°C, then purified by using a centrifugal tube (Millipore, Amicon Ultra, MWCO 100 kDa) to remove any unreacted PNIPAAm and salts. The supernatant was finally freeze-dried to obtain the FITC-BSA-NH_2/PNIPAAm nanoconjugate. Based on

MALDI-TOF measurements, there were on average 3.3 PNIPAAm chains covalently coupled to each BSA molecule.

Preparation of FITC-BSA-NH_2/PNIPAAm proteinosomes: FITC-BSA-NH_2/PNIPAAm proteinosomes were prepared according to a previously described protocol.[3] Briefly, 1.5 mg of PEG-bis(N-succinimidyl succinate) cross-linker was added to 60 µL of a 4.0 mg mL^{-1} FITC-BSA-NH_2/PNIPAAm solution in 50 mM carbonate buffer (pH 8.5), immediately followed by the addition of 1 mL of 2-ethyl-1-hexanol. The mixture was gently hand-shaken for 10s to produce a protein-polymer stabilized water-in-oil emulsion (aqueous/oil volume fraction, Φ_w, of 0.06). The cross-linker was allowed to react with the primary amine groups in BSA-NH_2 at the water-oil interface for 48h. After 48h sedimentation, the upper clear oil layer was discarded, and 1 mL of a 65% ethanol/water mixture added to dissolve the sediment. The solution was then dialyzed against 65%, 40% and 20% ethanol/water for 2h, then against Milli-Q water for 1 day to complete the transfer of the cross-linked proteinosomes in water.

Proteinosomes comprising encapsulated glucose oxidase (GOx) were prepared following the above protocol, except that the protein (20 µL, 30 mg mL^{-1}) was added to the aqueous FITC-BSA-NH_2/PNIPAAm solution before mixing with the oil phase. Alternatively, proteinosomes containing FITC-labelled GOx were prepared by using non-fluorescently labelled BSA-NH_2/PNIPAAm nanoconjugates. The aqueous proteinosome suspensions were gently centrifuged to remove any free protein. Typically, 200 µL of proteinosome suspension was centrifuged for 1 min at 60 ×g, then for a further 1 min at 390 ×g. 80 µL of the supernatant were removed and replaced with 80 µL Milli-Q water. The procedure was repeated at least 3 times.

Proteinosome-mediated pH decrease in fatty acid micelle coacervate suspensions: The pH of a freshly-prepared suspension of fatty acid micelle coacervate micro-droplets at 32 °C was adjusted to 10.0 by addition of 1M NaOH. Titration studies at different relative protocell density ratios were performed as follows: 4, 20, 40, or 60 µL of a GOx-containing proteinosome suspension were added to the micelle coacervate dispersion to reach a final volume of 200 µL and a final proteinosome : coacervate number density ratio of 1:140, 1:26, 1:12 and 1:7, respectively. To these mixtures were added 2 µL of a 1M glucose stock solution to reach a final glucose concentration of 10 mM, and the pH was monitored after ~30s of equilibration using a calibrated pH metre (Mettler Toledo) under continuous gentle magnetic stirring at 32 °C.

Titration studies at different glucose concentrations were performed as follows. 40 µL of a GOx-containing proteinosome suspension were added to the myristic acid micelle coacervate dispersion to reach a final volume of 200 µL and a final proteinosome : coacervate number density ratio of 1:12. To these mixtures were added 2 µL of a 100 mM glucose stock solution or 1, 2 or 4 µL of a 1M glucose stock solution to reach a final glucose concentration of 1, 5, 10 or 20 mM, respectively, and the pH was monitored after ~30s of equilibration under continuous gentle magnetic stirring at 32 °C. Typically, pH values were recorded every minute from t = 0 to t = 15 min, then every 15 minutes from t = 15 min to t = 60 min, then every hour from t = 60 min to t = 300 min, then values were acquired less frequently until the pH had stabilized to a final plateau value.

Turbidity values of the mixed proteinosome/coacervate suspensions (1:12 number density ratio) during the proteinosome-mediated pH decrease were acquired at 700 nm under continuous gentle stirring at 32°C using a UV-vis spectrophotometer (PerkinElmer) to confirm the disassembly of droplets (high turbidity) into vesicles (low turbidity).

Proteinosome-mediated disassembly of micelle coacervate micro-droplets: GOx-containing FITC-proteinosome/coacervate droplet dispersions were produced at pH ~ 9.2, T = 32 °C and a proteinosome : coacervate number density ratio of 1:12. 10 mM of glucose were added to the mixture, which was then rapidly (t < 2 min) loaded between a glass coverslip and a glass slide sealed together with a double-sided tape spacer (SecureSeal™ imaging spacer, Sigma-Aldrich). The droplets were allowed to settle for ~2 min before observations.

Vesicle self-assembly in proteinosomes: Mixed proteinosome/coacervate dispersions were produced at a proteinosome : coacervate number density ratio of 1:12 by adding 20 µL of a FITC-proteinosome dispersion to 80 µL of a Nile Red doped micelle coacervate droplets suspension freshly prepared at pH ~ 9.5 and 32 °C ([Nile Red] = 1 µM). 1 µL of a 1M glucose stock solution was added, and the dispersions were left to react under gentle magnetic stirring at 32 °C for 4 h, and then loaded between a glass slide and coverslip for imaging. Self-assembly of fatty acid vesicles in the proteinosomes was monitored by confocal fluorescence microscopy. Image analysis was performed with ImageJ, while 3D reconstruction of z-stacked acquisitions was done with Icy.

Two control experiments were also performed to investigate intra-proteinosome vesicle self-assembly under different conditions: (i) the pH of the mixed proteinosome/coacervate dispersions was rapidly decreased to ~8.5 by addition of 0.5 µL of 1M HCl (referred to as "pH jump"); and (ii) the pH of the mixed proteinosome/coacervate dispersions was gradually decreased by addition of 20 µL of 25 mM HCl in 1 µL steps added every 2 minutes under gentle magnetic stirring at 32 °C ("pH steps"). The final mixtures were loaded between a glass slide and a coverslip for confocal fluorescence microscopy imaging.

Selective payload transfer via proteinosome-mediated droplet-to-vesicle transition: Myristic acid micelle coacervate microdroplet dispersions containing 250 µM α-dansyl-L-arginine, 50 µM RITC-Mb, 50 µM RITC-HRP, 50 µM RITC-ALP or 2 µM TAMRA-*ss*DNA were produced at 32 °C and pH ~ 9.5 by adding 1-5 µL of aqueous stock solutions of the solutes to freshly-made micelle coacervate droplets to reach a final volume of 80 µL. 20 µL of GOx-containing FITC-proteinosomes were added to these dispersions to reach a final volume of 100 µL and a proteinosome : coacervate droplet number density ratio of 1:12. The pH decrease was initiated by the addition of 1 µL of a 1M aqueous stock solution of glucose (final [glucose] = 10 mM), and the mixture was kept at 32 °C for 4 hours under gentle magnetic stirring. After 4 hours, vesicles were formed as observed by a reduction in the turbidity of the sample, and the mixtures were loaded between a glass slide and a coverslip for microscopy imaging. Alternatively, for control experiments, 100 µL of GOx-containing proteinosome/coacervate droplet dispersions (proteinosome : coacervate number density = 1:12) were produced in the absence of any solute, and then allowed to react for 4 hours at 32 °C with 10 mM glucose to produce a proteinosome/vesicle dispersion. 1-5 µL of aqueous solute stock solutions were then added and the samples imaged by optical and fluorescence microscopy.

RITC- or TAMRA-tagged solutes were observed by optical and confocal fluorescence microscopy using the appropriate excitation wavelength and emission filters. In contrast, α-dansyl-L-arginine could not be imaged by confocal fluorescence microscopy due to the low excitation wavelength of 350 nm that was incompatible with the confocal set-up. Instead, samples containing this solute were imaged by epifluorescence microscopy by using a bandpass filter to select the appropriate excitation wavelength (340-380 nm) and cut-off filter (400 nm).

Enzymatic activity of HRP-containing vesicles: The catalytic activity of HRP-containing fatty acid vesicles produced by proteinosome-mediated disassembly of myristic acid micelle coacervate droplets was compared to control experiments where HRP was added to the external solution. Proteinosome/coacervate droplet dispersions were produced at pH ~ 9.5 and 32 °C, and at a proteinosome : coacervate number density ratio of 1:12 in the presence or absence of 20 nM HRP. 10 mM of glucose were then added and the mixture stirred at 32 °C for 5 hours. In samples prepared in the absence of the enzyme in the initial populations, 20 nM HRP was added. 50 µL of the final mixtures were loaded in a 96-well plate, and the enzyme reaction was initiated by injecting 5 µL of a stock aqueous solution of Amplex Red (500 µM). The fluorescence intensity of the oxidised product resorufin was monitored over time at 590 nm with an excitation at 540 nm by using a CLARIOstar plate reader (BMG Labtech). Triplicates were performed and the average and standard deviation of each time-dependent fluorescence intensity evolution was plotted.

Fluorescence-activated cell sorting (FACS): *Internalization of guest proteinosomes in host micelle coacervate micro-droplets.* Dispersions of FITC-labelled GOx-containing proteinosomes, unlabelled myristic acid micelle coacervate micro-droplets, or mixed proteinosome/coacervate populations at a proteinosome : coacervate number density ratio of 1:12 were analysed by FACS using a NovoCyte flow cytometer (ACEA Biosciences, Inc.) operated at a low pressure with a 100 µm sorting nozzle. 2D dot plots of the FSC and SSC light were determined for a total of 20,000 particles in the single or binary populations. The green fluorescence intensity emitted by individual protocells was monitored by excitation and detection with a 488 nm blue laser and a 530 ± 30 nm filter, and plotted as histograms of the numbers of counts for gated populations of coacervates, proteinosomes or vesicles against their corresponding green (FITC-A) fluorescence intensity. Data analysis was performed on FlowJo 10.3 software.

Fluorescence-activated cell sorting (FACS): *Proteinosome-mediated micelle coacervate disassembly.* GOx-containing FITC-proteinosomes/coacervate droplets dispersions were produced at pH ~ 9.2 and 32 °C, and with a proteinosome : coacervate number density ratio of 1:12. 10 mM of glucose were added to the mixture, and 2D plots of the FSC and SSC light were acquired at different times after addition of glucose. The counts of each population were extracted from the 2D dot plots by using appropriate gating to select only the counts associated to a specific population. Counts of the fatty acid micelle coacervate droplets were plotted before the vesicles started to form because the two populations partly overlapped in the 2D dot plots.

Optical and fluorescence microscopy studies: Bright-field and epifluorescence images were acquired using a Leica DMI3000B inverted microscope equipped with a ×20 objective lens, 0.7 NA. Confocal fluorescence microscopy observations were performed using a Leica SP5-II confocal laser scanning microscope attached to a Leica DMI6000 inverted epifluorescence microscope equipped with a 65 mW Ar Laser (488 nm for FITC excitation, 20% power) and a 20 mW solid state yellow laser (561 nm for RITC or Nile Red excitation, 20% power). Detection bands were set at 500-550 nm (FITC) and 575-700 nm (RITC and Nile Red). Droplets were allowed to settle for ~2 min before observations.

Measurement of protocell number densities: Fatty acid micelle coacervate and proteinosome number densities were determined using epifluorescence microscopy. 1 µL aliquot of a Nile Red-doped micelle coacervate or FITC-labelled proteinosome suspension was loaded between a glass slide and a coverslip, then imaged by epifluorescence microscopy. The number of protocells were counted from the recorded images. On average, the number densities of micelle coacervate micro-droplets and proteinosomes were $4.5 \pm 1 \times 10^6$ counts/mL and $1.5 \pm 0.8 \times 10^6$ counts/mL, respectively.

Sequestration efficiency and statistical distribution of proteinosomes in micelle coacervate micro-droplets: Mixed coacervate/proteinosome dispersions at a final proteinosome : coacervate droplet number density ratio of 1:140, 1:26, 1:12 or 1:7 were produced by adding 2, 10, 20 or 30 µL of a FITC-proteinosome suspension to a Nile Red-doped myristic acid micelle coacervate dispersion to reach a final volume of 100 µL. The mixtures were loaded between a glass slide and a coverslip and imaged by epifluorescence microscopy. The sequestration efficiency (in %) was determined by counting the number of proteinosomes internalized in coacervate droplets on the acquired images by using *ImageJ*, and normalizing it to the total number of proteinosome counted. The number of micelle coacervate droplets containing 1, 2 or 3 or more proteinosomes were also counted, and normalized to the total number of proteinosome-containing droplets counted to give the statistical distribution of proteinosomes within the micelle coacervate micro-droplets. Experiments were repeated three times and the average sequestration and statistical distribution values and associated standard deviations reported.

Determination of equilibrium partition constants in fatty acid micelle coacervate micro-droplets: The equilibrium partition coefficient (K) of different solutes in the myristic acid micelle coacervate phase was determined from the equation K = $[solutes]_{in}/[solute]_{out}$, where $[solute]_{in}$ and $[solute]_{out}$ were the concentration of the solute in the micelle coacervate phase and the continuous aqueous phase, respectively. Typically, 50 µL of aqueous stock solution of fluorescently-labelled solutes at ~ 5 mg mL^{-1} were added to 1 mL of a fatty acid micelle coacervate droplets dispersion, and the mixture was left to settle at 32 °C. After 10-24h, macroscopic phase separation of the coacervate droplets had occurred and resulted in the formation of an upper bulk coacervate phase (d = 940 g L^{-1} < d_{water}) and a lower dilute aqueous phase. The concentration of solute in both phases was measured by UV-vis spectroscopy at the maximum of absorption of the dyes (350 nm for dansyl, 488 nm for FITC and 557 nm for RITC) by using a Perkin-Elmer Lambda 35 spectrophotometer. Typically, 20 µL of the upper phase were diluted in 480 µL of water to disassemble the coacervate, and 250 µL of the lower aqueous phase were diluted into 250 µL of water before the UV-vis measurements. Experiments were repeated three times and the average K values and standard deviations reported.

Preparation of fluorescently-labelled proteins: A protein solution (4 mg mL^{-1}) was prepared by dissolving the freeze-dried protein powder in 1 mL of 0.5M carbonate buffer at pH = 9.0. An aliquot of a freshly-prepared anhydrous DMSO solution of rhodamine (RITC) or fluorescein (FITC) isothiocyanate (10 mg mL^{-1}) was added dropwise to the protein solution at a final fluorophore:protein molar ratio of ca. 10:1. The reaction mixture was kept at room temperature in the dark for 4 hours, then purified by size exclusion chromatography using a Sephadex G-25 resin (Sigma-Aldrich) eluted with milli-Q water. The concentration of the fluorescently-labelled proteins in the collected fractions was determined by UV-visible spectrophotometry according to the relationship: [protein] = $(A_{280} – w \times A_{max,dye}) / \varepsilon_{protein}$, where A_{280} and $A_{max,dye}$ were the absorbances at 280 nm and at the maximum of absorption of the fluorophores respectively (488 nm for FITC, 552 nm for RITC), *w* the correction factor to account for the dye absorption at 280 nm (0.30 (FITC), 0.34 (RITC)), and $\varepsilon_{protein}$ the extinction coefficient of the protein (1.67 mL mg^{-1} cm^{-1} (GOx), 0.656 mL mg^{-1} cm^{-1} (HRP), 0.71 mL mg^{-1} cm^{-1} (ALP), 0.82 mL mg^{-1} cm^{-1} (Mb), 1.83 mL mg^{-1} cm^{-1} (CAB)). The dye:protein final molar ratio was determined from the ratio $(A_{max,dye} / \varepsilon_{dye})$ / ([protein] (mg mL^{-1}) / $M_{protein}$), where ε_{dye} was the molar extinction coefficient of the dyes at their maximum of absorption (68,000 mol^{-1} L cm^{-1} (FITC), 65,000 mol^{-1} L cm^{-1} (RITC)), and $M_{protein}$ the molar mass of the protein (80,000 g mol^{-1} (GOx), 40,000 g mol^{-1} (HRP), 150,000 g mol^{-1} (ALP), 16,700 g mol^{-1} (Mb), 30,000 g mol^{-1} (CAB)). Typically, the average dye:protein molar ratio was *ca*. 2:1.

Supplementary References

[1] Garenne, D. *et al.* Clouding in fatty acid dispersions for charge-dependent dye extraction. *J. Colloid Int. Sci.* **468**, 95-102 (2016).
[2] Garenne, D. *et al.* Sequestration of proteins by fatty acid coacervates for their encapsulation within vesicles. *Angew. Chem. Int. Ed.* **55**, 13475-13479 (2016).
[3] Huang, X. *et al.* Interfacial assembly of protein–polymer nano-conjugates into stimulus-responsive biomimetic protocells. *Nat. Commun.* **4**, 2239 (2013).
