# Supplementary material for: Antagonistic chemical coupling in self-reconfigurable host–guest protocells
Source: Nat Commun. 2018 Sep 7;9:3652. doi: 10.1038/s41467-018-06087-3 (PMC6128866; doi:10.1038/s41467-018-06087-3)
Supplement: Supplementary file 2 — Description of Additional Supplementary Files [file 41467_2018_6087_MOESM2_ESM.pdf]

Description of Additional Supplementary Files

Supplementary Movie 1
Description: Epifluorescence microscopy video showing wetting and capture of an individual proteinosome labelled with FITC (green) by a single micelle coacervate droplet doped with Nile Red (red) and subsequent coalescence with a larger coacervate droplet. Movie is shown at real-time speed at 7 frames per second. Total duration of recording was 19 seconds in real time. Scale bar = 50 µm.

Supplementary Movie 2
Description: Optical microscopy video showing proteinosome guest-mediated disassembly of a host fatty acid coacervate droplet. Movie is shown at ×100 of real-time speed at 10 frames per second. Total duration of recording was 20 minutes in real time. Scale bar = 50 µm.

Supplementary Movie 3
Description: Confocal fluorescence (left panel) and corresponding optical microscopy (right panel) videos showing the formation of elongated fatty acid protrusions at the coacervate/water interface during proteinosome guest-mediated disassembly of a host coacervate droplet. The tubular vesicles transform into spherical vesicles either directly by self-assembly in the external solution, or indirectly via diffusion of free fatty acid molecules into the proteinosome interior. Movie is shown at ×150 of real-time speed at 5 frames per second. Total duration of recording was 20 minutes in real time. Scale bar = 50 µm.

Supplementary Movie 4
Description: Optical microscopy video showing self-induced capture of a urease/GOx-containing proteinosome via urease-mediated transformation of fatty acid vesicles into micelle coacervate droplets in the presence of urea (20 mM). The droplets gradually coalesce at the surface of the proteinosome to form a proteinosome-in-coacervate host-guest protocell. Movie is shown at ×100 of real-time speed at 10 frames per second. Total duration of recording was 24 minutes in real time. Scale bar = 50 µm.
